# *Bifidobacterium pseudocatenulatum* CECT 7765 Reduces Obesity-Associated Inflammation by Restoring the Lymphocyte-Macrophage Balance and Gut Microbiota Structure in High-Fat Diet-Fed Mice

**DOI:** 10.1371/journal.pone.0126976

**Published:** 2015-07-10

**Authors:** Angela Moya-Pérez, Alexander Neef, Yolanda Sanz

**Affiliations:** Microbial Ecology, Nutrition & Health Research Group, Institute of Agrochemistry and Food Technology, National Research Council (IATA-CSIC), Valencia, Spain; College of Tropical Agriculture and Human Resources, University of Hawaii, UNITED STATES

## Abstract

**Background/Objectives:**

The role of intestinal dysbiosis in obesity-associated systemic inflammation via the cross-talk with peripheral tissues is under debate. Our objective was to decipher the mechanisms by which intervention in the gut ecosystem with a specific *Bifidobacterium* strain reduces systemic inflammation and improves metabolic dysfunction in obese high-fat diet (HFD) fed mice.

**Methods:**

Adult male wild-type C57BL-6 mice were fed either a standard or HFD, supplemented with placebo or *Bifidobacterium pseudocatenulatum* CECT 7765, for 14 weeks. Lymphocytes, macrophages and cytokine/chemokine concentrations were quantified in blood, gut, liver and adipose tissue using bead-based multiplex assays. Biochemical parameters in serum were determined by ELISA and enzymatic assays. Histology was assessed by hematoxylin-eosin staining. Microbiota was analyzed by 16S rRNA gene pyrosequencing and quantitative PCR.

**Results:**

*B*. *pseudocatenulatum* CECT 7765 reduced obesity-associated systemic inflammation by restoring the balance between regulatory T cells (Tregs) and B lymphocytes and reducing pro-inflammatory cytokines of adaptive (IL-17A) and innate (TNF-α) immunity and endotoxemia. In the gut, the bifidobacterial administration partially restored the HFD-induced alterations in microbiota, reducing abundances of Firmicutes and of LPS-producing Proteobacteria, paralleled to reductions in B cells, macrophages, and cytokines (IL-6, MCP-1, TNF-α, IL-17A), which could contribute to systemic effects. In adipose tissue, bifidobacterial administration reduced B cells whereas in liver the treatment increased Tregs and shifted different cytokines (MCP-1 plus ILP-10 in adipose tissue and INF-γ plus IL-1β in liver). In both tissues, the bifidobacteria reduced pro-inflammatory macrophages and, TNF-α and IL-17A concentrations. These effects were accompanied by reductions in body weight gain and in serum cholesterol, triglyceride, glucose and insulin levels and improved oral glucose tolerance and insulin sensitivity in obese mice.

**Conclusions:**

Here, we provide evidence of the immune cellular mechanisms by which the inflammatory cascade associated with diet-induced obesity is attenuated by the administration of a specific *Bifidobacterium* strain and that these effects are associated with modulation of gut microbiota structure.

## Introduction

Obesity has become a major global health challenge due to its increasing prevalence and the associated health risks [1]. Obesity results from a positive energy imbalance associated with alterations in the metabolic-immune axis, leading to chronic low-grade inflammation [2]. The inflammatory process is characterized by infiltration of macrophages and lymphocytes in the adipose tissue and other peripheral organs. This is accompanied by an imbalance in the cytokine network with increased production of pro-inflammatory cytokines and adipokines, which contribute to associated metabolic dysfunctions, such as insulin resistance [3]. White adipose tissue has been considered the main contributor to systemic inflammation in obesity. Inflammation of this tissue is mediated by increased infiltration of macrophages and the ratio of “classically activated macrophages” (M1) to “alternatively activated macrophages” (M2), which produced pro-inflammatory (e.g. TNF-α, IL-1β and IL-6) and anti-inflammatory (IL-10 and IL-4) cytokines, respectively [4]. Obese adipose tissue is also characterized by increased infiltration of B cells and shifts in CD8+/CD4+ T lymphocytes and reduced CD4+ regulatory T (Treg) cells [5–7]. The adipose tissue is also thought to provide an immediate source of free fatty acids and pro-inflammatory cytokines (e.g. TNF-α, IL-6, and IL-1β) to the liver, which may trigger hepatic inflammation [8,9]. A connection between visceral fat and intestinal inflammation has also been identified in Crohn disease [8,10]. However, whether adipose tissue or intestinal inflammation is the origin of systemic inflammation associated with diet-induced obesity is under debate [8,9,11].

The gut microbiota interacts and regulates multiple host metabolic and immunologic pathways, some of which could mediate the communication between the gut and other distal organs, such as the liver [8,12,13] and the adipose tissue [8,14]. Studies in animal models have provided evidence for a role of the microbiota in bodyweight regulation by influencing energy extraction, inflammation and neuroendocrine secretions [15]. Thus, intentional manipulation of the gut microbiota could be an additional strategy to reduce the incidence of obesity and its comorbidities. In human observational studies, reduced numbers of *Bifidobacterium* spp. have been associated with the development of overweight [15]. Moreover, dietary interventions based on the use of potential probiotics or prebiotics, leading to increase the numbers of *Bifidobacterium* spp. in the gut have demonstrated pre-clinical efficacy, by ameliorating some of the obesity-associated metabolic alterations and immune dysfunction in animal models [16,17]. Nevertheless, the mechanisms by which specific intestinal bacteria may reduce systemic and peripheral tissue inflammation associated with diet-induced obesity are largely unknown, particularly regarding their potential ability to regulate components of adaptive immunity.

This study is based on the hypothesis that *B*. *pseudocatenulatum* CECT 7765 can reduce HFD-induced intestinal inflammation, thereby helping to ameliorate systemic and peripheral tissue inflammation and to improve metabolic function. To this end, we orally administered *B*. *pseudocatenulatum* CECT 7765 to mice with HFD-induced obesity and monitored its effects on the intestinal ecosystem and on immune cell infiltration and inflammatory and metabolic markers in serum and different tissues (adipose tissue, gut, and liver).

## Methods and Materials

### Bacterial strain and culture conditions

*Bifidobacterium pseudocatenulatum* CECT 7765 was originally isolated from stool of breast-fed infants and identified by sequencing amplified 16S rDNA regions as described previously [17]. This strain was initially selected from among other isolates as a potential probiotic for preventing obesity-associated inflammation on the basis of its anti-inflammatory properties in a macrophage cell line [17]. The bacterial strain was grown in MRS broth (Scharlau, Barcelona, Spain) supplemented with 0.05% (w/v) cysteine (MRS-C) (Sigma, St. Louis, MO), and incubated at 37°C for 24 h under anaerobic conditions (AnaeroGen, Oxoid, Basingstoke, UK). Cells were harvested by centrifugation (2,000 g for 15 min), washed twice in phosphate buffered saline (PBS, 130 mM sodium chloride, 10 mM sodium phosphate, pH 7.4), and re-suspended in 10% skimmed milk for oral administration to mice. Aliquots of these suspensions were frozen in liquid nitrogen and stored at -80°C until used. The number of live cells after freezing and thawing was determined by colony-forming unit (CFU) counting on MRS-C agar after 48 h incubation. For each strain tested, more than 90% cells were alive upon thawing and no significant differences were found during storage time (2 months). One fresh aliquot was thawed for every new experiment to avoid variability in bacterial viability.

### Animals, diets and experimental design

Adult (aged 6–8 week) male wild-type C57BL-6 mice were purchased from Charles River Laboratories (L'Arbresle Cedex, France). In the adaptation period (7 days), animals of each experimental group were housed together in a stainless-steel cage in a temperature-controlled (23°C) room with a 12-h light/dark cycle and 40–50% relative humidity and were fed a standard diet (SD) *ad libitum*. Then, mice were randomly divided into four groups (n = 10 mice per group) as follows: (1) control mice receiving a SD plus placebo (10% skimmed milk) (SD group); (2) obese mice receiving a high-fat diet (HFD) plus placebo (HFD group); (3) control mice receiving SD and a daily dose of 1 x 10^9^ CFU *B*. *pseudocatenulatum* CECT 7765 (SD+Bif group); (4) obese mice receiving HFD and a daily dose of 1 x 10^9^ CFU *B*. *pseudocatenulatum* CECT 7765 (HFD+Bif group) by oral gavage.

This regime was maintained for fourteen weeks. To induce obesity, mouse groups 2 and 4 were switched from the SD (CA.170481—AIN-76A Purified Diet-Rats/Mice, Harlan Laboratories, Madison, WI 53744–4220) administered during the adaptation period to a HFD (TD.06414—Adjusted Calories Diet—60/Fat [lard 31% and soybean oil 3%] Harlan Laboratories, Madison, WI 53744–4220) for 14 weeks. The HFD provided 18.4% kcal as protein, 21.3% kcal as carbohydrate and 60.3% kcal as fat (5.1 kcal/g), whereas the SD provided 18.8% kcal as protein, 68.8% kcal as carbohydrate and 12.4% kcal as fat (3.8 kcal/g). Mice had free access to water and feed.

Experiments were carried out *in strict accordance with the recommendations in the Guide for the Care and Use of Laboratory Animals of the* University of Valencia (Central Service of Support to Research [SCSIE], University of Valencia, Spain) and the study protocol was approved by its Ethical Committee.

Body weight was measured once a week. Faecal samples were collected a few days before slaughter. After 14 weeks of dietary intervention animals were fasted for 16 h, anaesthetized with isoflurane and sacrificed by cervical dislocation. For analysis of hormonal and metabolic parameters, blood samples were collected in tubes, two for each animal, containing EDTA, one of them was centrifuged (2,000 g for 10 min at 4°C) and the supernatant (serum) was kept at -20°C, the other one was used for blood cell flow cytometry analysis. The left parts of liver and white adipose tissue (epididymal) and small intestine (ileo) were suspended in phosphate buffered saline solution (PBS, 130 mM sodium chloride and 10 mM sodium phosphate, pH 7.4) and kept at 4°C until processing for further flow cytometry analysis. Moreover, liver and white adipose tissue (epididymal) sections were fixed in 10% neutral formalin buffered solution for histological analysis. The right parts of liver, white adipose tissue (epididymal) and small intestine (ileo) were suspended in RNA Later (Qiagen, Madrid, Spain) and kept at -80°C for cytokine quantification.

### Hormonal and metabolic parameter analyses

Serum leptin concentration was determined by the Assay Max Mouse Leptin ELISA kit (Assay pro, LLC; Ireland) with a sensitivity threshold of 0.3 ng/ml. Biochemical parameters were also quantified in serum using enzymatic kits for glucose (Glucose Liquid Kit), cholesterol (Cholesterol Liquid kit) and triglycerides (Triglyceride Liquid kit, all from Química Analítica Aplicada SA, Spain), according to the manufacturer’s instructions. Insulin was measured using a Rat/Mouse ELISA kit (Merck Millipore, Germany) with a sensitivity threshold of 0.2 ng/ml. The enzymes aspartate amino-transferase (AST) and alanine aminotransferase (ALT) were measured using two assay kits from BioVision (Milpitas, USA) both with a sensitivity threshold of 10 mU.

### Oral glucose tolerance test (GTT) and intraperitoneal insulin tolerance test (ITT)

The GTT was performed *in vivo* after 13 weeks of dietary intervention. The GTT was performed after 6 h of food deprivation, after which 2.0 g/kg body weight glucose was administered orally. Blood samples were taken with heparinised capillary tubes from the saphenous vein at baseline and 15, 30, 45, 60 and 120 minutes after oral glucose administration. Plasma glucose levels were analysed with glucose test strips (AscensiaEsyfill, Bayer, Tarrytown, NY; USA) and a glucometer (Ascensia VIGOR, Bayer Tarrytown), with a detection level ranging from 30 to 550 mg glucose/dl. The area under the glucose curve (AUC) was estimated by plotting the glucose concentration (mg/dl) versus time (min).

The ITT was conducted after 6 h of food deprivation, followed by intraperitoneal injection of 1.5 U/kg body weight insulin (HumulinR, Eli Lilly,Egypt) and, then, glucose was measured as described for the GTT.

### Histology of liver and white adipose tissues

Paraffin-embedded tissues were sectioned to a thickness of 4–5 μm and fixed to glass slides. Slides were deparaffinised and stained with hematoxylin-eosin. The severity of steatosis was determined in100 hepatocytes of two liver tissue sections per mouse and scored as follows: grade 0 when fat was not detected in hepatocytes; grade 1 when fat occupied less than 30% of hepatocytes; grade 2 when fat occupied between 30 and 60% of hepatocytes; grade 3, when fat occupied more than 60% of hepatocytes as described previously [17].

Adipocyte cell sizes were measured in 100 cells of two sections of epididymal adipose tissue per mouse and adipocyte cell sizes were expressed as area ranges using the following ranges: <2000, 2000–4000, 4000–6000 and 6000–7000 μm^2^, as described previously [17]. All parameters were measured in a NIKON Eclipse 90i Microscope, using the NIS Elements BR 2.3 basic research software (Kingston, Surrey, KT25PR, England). All histological analyses were conducted blind by an experienced histologist.

### Cytokine quantification in serum and different tissues

Frozen tissues (small intestine, liver and adipose tissue) were weighed and homogenized using a Tissue Ruptor (Qiagen, Madrid, Spain) in RIPA buffer (1 × solution, 150 mM NaCl, 1.0% IGEPAL CA-630, 0.5% sodium deoxycholate, 0.1% SDS, and 50 mM Tris, pH 8.0) (Sigma, Madrid, Spain). This method enables efficient cell lysis and protein solubilisation while avoiding protein degradation and interference with the proteins immunoreactivity. These samples were incubated for 10 min, homogenised with Tissue Ruptor (Qiagen, Madrid, Spain) at 4°C for 1 min and centrifuged at 10,000×*g*, at 4°C for 5 min. Supernatants were stored at -80°C until analysed.

For cytokine quantification, the Mouse/Rat Basic Kit Flow Cytomix BMS8440FF combined with a bead-based Analyte Detection Assay for each cytokine (FlowCytomix Simplex Kits; eBioscience, Affymetrix Company, Vienna, Austria) were used. Cytokines were quantified with a FACS Canto cytometer (Becton, Dickinson and Company, NJ). The following cytokines and chemokines were analysed: IFN-γ, IL-6, TNF-α, IL-10, MCP-1, IL-18, IL-4, IL-1β and IP-10 (eBioscience, Affymetrix Company, Vienna, Austria). Sensitivity thresholds for each cytokine were: 6.5 pg/ml for IFN-γ, 2.2 pg/ml for IL-6, 2.9 pg/ml for TNF-α, 13.3 pg/ml for IL-10, 42 pg/ml for MCP-1, 116.9 pg/ml for IL-18, 0.7 pg/ml for IL-4, 34.3 pg/ml forIL-1β and 9.8 pg/ml for IP-10. Also IL-17 was analysed using a Mouse IL-17A ELISA kit (eBioscience, Affymetrix Company, Vienna, Austria) with a sensitivity threshold of 4 pg/ml. Data are presented as pg cytokine/g of tissue weight or ml for serum samples.

### Lymphocyte and macrophage populations and TLR4 and TLR2 expression

Epididymal white adipose tissue, a well-accepted source of intra-abdominal fat, mesenteric lymph nodes (MLN) from small intestine (ileo) and liver were used for immune-cell analysis by flow cytometry. For adipose tissue, visible vessels and connective tissue were carefully removed and the tissue was washed extensively with PBS, minced with fine scissors and digested with 0.1% collagenase from *Clostridium histolyticum* C6885 (Sigma Aldrich) at 37°C for 30 minutes. Liver and MLN were also cut in small pieces. Afterwards, cut tissues were passed through 40 μm mesh filters and washed in FACS buffer (PBS with 0.5% BSA and 2 mM EDTA). The suspension in the FACS buffer was left to settle in a conical tube for 5–6 minutes and then centrifuged at 400 g for 5 min and the pelleted cells were analyzed. To evaluate lymphocyte populations in peripheral blood, 100 μl blood was re-suspended in antibody solution and incubated for 30 min in darkness; then, 2 ml cell lysis buffer for red blood cells (160 mmol NH, 0.1 mmol EDTA, 12 mmol NaHCO) were added, and the samples were centrifuged (300 g for 5 min). Afterwards, the cell pellet was washed twice with 2 ml FACS buffer (PBS, 0.03% sodium azide, 0.1% bovine serum albumin) and centrifuged again. Then, the cell pellet was re-suspended in 300 μl FACS buffer and analysed by flow cytometry [18].

Cells were stained with the following fluorescent dye-labelled mouse monoclonal antibodies: CD3-FITC, CD4-PE and CD8-APC for detection of total lymphocytes (CD3+) and the T helper (CD3+CD4+CD8-) and T cytotoxic (CD3+CD4-CD8+) subtypes; CD4-APC, CD25-PE and Foxp3-FITC for detection of regulatory T cells (CD4+CD25+CDFoxp3+); CD19-eFluor450 for detection of B cells (CD19+); and F/480-PE, CD11c-FITC and CD206-APC for detection of total macrophages (F4/80+), M1 macrophages (F4/80+CD11c+CD206-) and M2 macrophages (F4/80+CD11c-CD206+). The expression of TLR4 and TLR2 in the small intestine was determined using the antibodies CD282-FITC and CD284-PE, respectively. All conjugated antibodies were from BD Biosciences (San Jose, CA, USA) except for CD206 that was from BioLegend (San Diego, CA, USA) and they were used according to the manufacturer’s instructions. Flow cytometry analyses were performed on a BD LSRFortessa cytometer (Becton, Dickinson and Company, NJ). Data were analyzed using BD FACS DIVA Software v.7.0. For detection of B cells with CD19-eFluor450 flow cytometry analysis was performed on a BD FACSVerse cytometer (Becton, Dickinson and Company, NJ). Data were analyzed using BD FACS Suite Software v.1.0.3.2942.

### Detection of Gram-negative bacterial endotoxin

Serum samples were extracted and instantly frozen at -20°C in glass vials. Lipopolysaccharide (LPS) endotoxin was quantified using a commercially available Limulus Amebocyte Lysate (LAL) Chromogenic Endotoxin Quantitation Kit (Thermo Scientific, Rockford,IL), according to the supplier’s instructions and as described previously [19].

### Microbiota composition analyses by pyrosequencing of 16S rDNA amplicons and quantitative PCR (qPCR)

Stool samples were weighed, diluted 1:5 (w/v) in PBS (pH 7.2), homogenised by shaking in a vortex and stored at -80°C till analysed. Aliquots (200μl) of this dilution were used for DNA extraction using the QIAamp DNA stool Mini kit (Qiagen, Hilden, Germany) after an initial step to favour cell disruption mechanically by mixing (1:1) the cell suspension with sterile zirconia/silica beads (diameter, 0.1 mm) in a bead-beater during 2 minutes.

Extracted DNA was used to amplify the V3 to V5 regions of the 16S rRNA gene using the primers 340F 5´- CCTACGGGAGGCAGCAG -3´ and 926R 5´- CCGTCAATTYMTTTRAGT -3´, a primer optimized to detect bifidobacteria [20]. Pyrosequencing was carried out at LifeSequencing (Paterna, Spain) using primer A on a 454 Life Sciences Genome Sequencer FLX instrument (Roche, Basel, Switzerland) yielding in total 411,536 reads for the 32 sequenced samples.

For sequence and clustering analysis original reads were filtered by length (> 300 bp) and quality (average Phred value > 25, no ambiguities) and then for chimeras using the mothur package [21] resulting in 251,002 classifiable sequence reads with an average length of 513 bp. Reads were identified at phylum, family and genus level using the Ribosomal Database Project (RDP) multiclassifier tool at an 80% confidence level [22]. Sequences that had confidence values below 0.8 were assigned to a group of "unclassified" organisms on the respective next higher taxonomic level. BlastN comparisons were performed to identify sequences on the species and genus level using a database containing 9,701 16S rRNA gene reference sequences (SILVA 111 Living Tree Project dataset, www.arb-silva.de). Rarefaction curves and alpha-diversity indices (Sobs, Shannon, Simpson) were calculated with the mothur package [21]. For rarefaction curves 1000 randomizations were applied.

Similarity of microbiota among different samples was evaluated by cluster analysis on genus level using the following two types of analysis. Bray-Curtis distances between microbiota of individual samples were computed on the basis of the relative abundances of different genera and a heatmap was generated for 52 genera with abundances > 0.1% in at least one sample using the algorithm implemented in the "vegan" package for R (http://vegan.r-forge.r-project.org). Also, weighted UniFrac analysis was performed using QIIME [23] and the FastUniFrac tool at the URL http://unifrac.colorado.edu with 1289 sequence types with abundances of at least 10 reads that represented in total 65,104 reads. Read data are deposited at ENA under accession numbers ERR692069-ERR692100.

Bifidobacteria were also quantified using 16S rRNA gene-specific primers for this genus [24,25] by qPCR using LightCyclerH 480 SYBR Green I Master (Roche, USA) with an ABI PRISM 7000- PCR sequence detection system (Applied Biosystems, UK), as described previously [26].

### Statistical analyses

All data were analyzed using Graph Pad Prism software (LaJolla, CA). Data distribution was analyzed by the Kolmogorov-Smirnov normality test. For normally distributed data differences were determined with one or two-way ANOVA (as appropriate) and *post hoc* Bonferroni’s test. Non-normally distributed data were analyzed with the non-parametric Mann-Whitney *U* test. Statistically significant differences in taxon abundances obtained by pyrosequencing were determined using a permutation test (DAAG package version 1.18 for R). Correlations between variables were established with the Spearman rank correlation coefficient. In every case, *p*-values < 0.05 were considered statistically significant.

## Results

### *B*. *pseudocatenulatum* CECT 7765 reduced metabolic alterations in obese mice

The administration of *B*. *pseudocatenulatum* CECT 7765 significantly reduced relative body weight gain by approximately 30% (*p* = 0.002) in the HFD group at the end of the intervention, but did not modify body weight gain in the SD group (Table 1). After the intervention, the obese group supplemented with *B*. *pseudocatenulatum* CECT 7765 exhibited a slight reduction in the weight of epididymal adipose tissue compared to mice fed the HFD and placebo (*p* = 0.082) (Table 1). Hepatic steatosis and adipocyte hypertrophy was also reduced by *B*. *pseudocatenulatum* CECT 7765 in HFD-fed mice. The administration of *B*. *pseudocatenulatum* CECT 7765 to HFD-fed mice increased the number of small adipocytes (<2000 and 2000–4000 μm^2^) and reduced the largest ones (>6000 μm^2^) (Table in S1 File and Fig in S1 File). The bifidobacteria also reduced hepatic steatosis in the HFD group by increasing the number of hepatocytes with 0 and 1-grade steatosis and reducing those with 2 and 3-grade steatosis (Table in S2 File and Fig in S2 File). The administration of *B*. *pseudocatenulatum* CECT 7765 to the HFD group also led to a significant reduction in serum cholesterol (*p* = 0.039), triglyceride (*p* = 0.003) and glucose (*p*<0.001) concentrations by approximately 7, 50 and 30%, respectively, as compared to the levels reached in the HFD group receiving only placebo (Table 1). *B*. *pseudocatenulatum* CECT 7765 administration also reduced the HFD-increased leptin (*p* = 0.034) and fasting insulin levels (*p* = 0.038), (Table 1). Amylase activity and ALT were higher in the HFD group than in the SD group (*p*<0.001 and 0.064, respectively) and the bifidobacteria strain slightly reduced the serum levels (*p* = 0.075 and 0.082), but none of the changes was statistically significant (Table 1).

**Table 1:** Weight gain and serum hormonal and biochemical parameters in different mouse groups after 14 weeks of dietary intervention.

**Outcome measure**	**Experimental Groups**											
	**SD**		**HFD**		**SD+Bif**		**HFD+Bif**		***p*-value** (HFD vs. SD)	***p*-value** (SD+ Bif vs. SD)	***p*-value** (HFD+ Bif vs. HFD)	***p*-value** (HFD+ Bif vs. SD)
	Mean	*se*	Mean	*se*	Mean	*se*	Mean	*se*				
Body weight gain (%)	35.03	4.02	62.30	4.39	32.83	1.29	44.00	2.66	<0.001*	0.625	0.002*	0.204
Epididymal adipose tissue weight (%)	1.09	0.04	5.88	0.42	1.11	0.04	4.52	0.38	0.001*	0.175	0.082	0.004*
Cholesterol (mg/dl)	93.15	4.78	171.20	6.09	97.96	4.14	159.32	3.75	<0.001*	0.474	0.039*	<0.001*
Triglyceride (mg/dl)	102.21	8.28	151.91	13.84	98.39	8.31	70.83	5.04	0.022*	0.777	0.003*	0.045*
Glucose (mg/dl)	161.93	10.51	251.25	8.97	147.20	6.89	176.05	11.22	<0.001*	0.241	<0.001*	0.412
Insulin (ng/ml)	0.47	0.04	2.09	0.29	0.43	0.09	1.05	0.21	0.004*	0.234	0.038*	0.001*
Leptin (ng/ml)	9.29	0.37	36.53	2.70	8.84	0.62	29.78	2.57	<0.001*	0.596	0.034*	<0.001*
Amylase (U/ml)	0.91	0.01	1.08	0.02	0.89	0.02	0.99	0.04	<0.001*	0.395	0.075	0.045*
ALT (nmol/μl)	0.06	0.01	0.09	0.01	0.07	0.00	0.07	0.01	0.064	0.290	0.082	0.588

SD group: control mice receiving a standard diet (SD) plus placebo; HFD group: obese mice receiving a high-fat diet (HFD) plus placebo; SD+Bif group: control mice receiving SD and a daily dose of 1 x 10^9^ CFU *B*. *pseudocatenulatum* CECT 7765; HFD+Bif group: obese mice receiving HFD and a daily dose of 1 x 10^9^ CFU *B*. *pseudocatenulatum* CECT 7765 by gavage during 14 weeks. Data are expressed as mean and standard error of the mean (*se*) of each mouse group (n = 10 per group).

*Significant differences were established by ANOVA and *post hoc* Bonferroni’s test at *p*<0.050.

**Fig 1:**
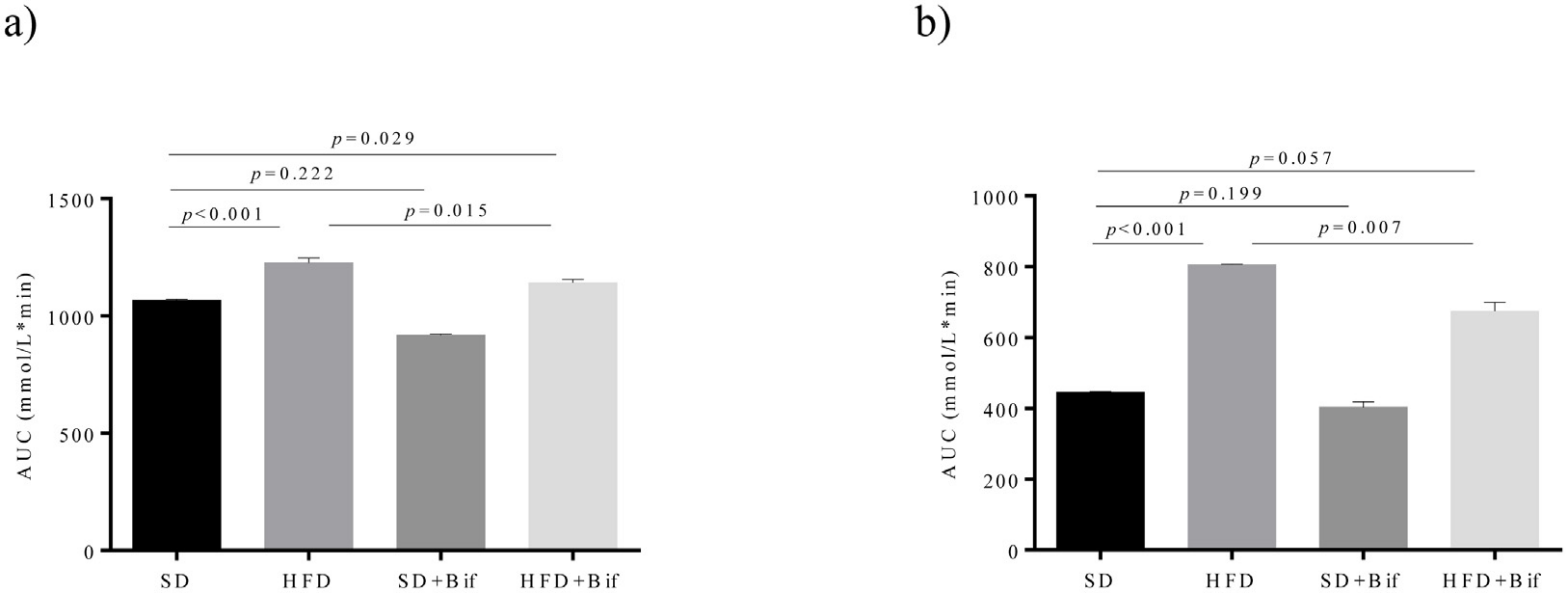
Oral glucose tolerance (a) and insulin sensitivity (b) in different mouse groups after 14 weeks of dietary intervention. SD group: control mice receiving a standard diet (SD) plus placebo; HFD group: obese mice receiving a high-fat diet (HFD) plus placebo; SD+Bif group: control mice receiving SD and a daily dose of 1 x 10^9^ CFU *B*. *pseudocatenulatum* CECT 7765; HFD+Bif group: obese mice receiving HFD and a daily dose of 1 x 10^9^ CFU *B*. *pseudocatenulatum* CECT 7765 by gavage during 14 weeks (n = 10). Data are expressed as means of area under curve (AUC) from the plots of glucose concentration (mmol/L) *versus* time (min) (standard errors in bars). Statistically significant differences were determined by ANOVA with Bonferroni’s *post hoc* test a *p*< 0.050.

*B*. *pseudocatenulatum* CECT 7765 also improved oral glucose tolerance (Fig 1a) and insulin sensitivity in the HFD group (Fig 1b). The bifidobacterial strain administration reduced the increase of area under the curve of the GTT test (*p* = 0.015; Fig 1a) and the area under the curve of the ITT test (*p* = 0.007; Fig 1b). *B*. *pseudocatenulatum* CECT 7765 administration to control mice did not modify these parameters.

### *B*. *pseudocatenulatum* CECT 7765 reduced B lymphocyte and macrophage infiltration in adipose tissue of obese mice

**Fig 2:**
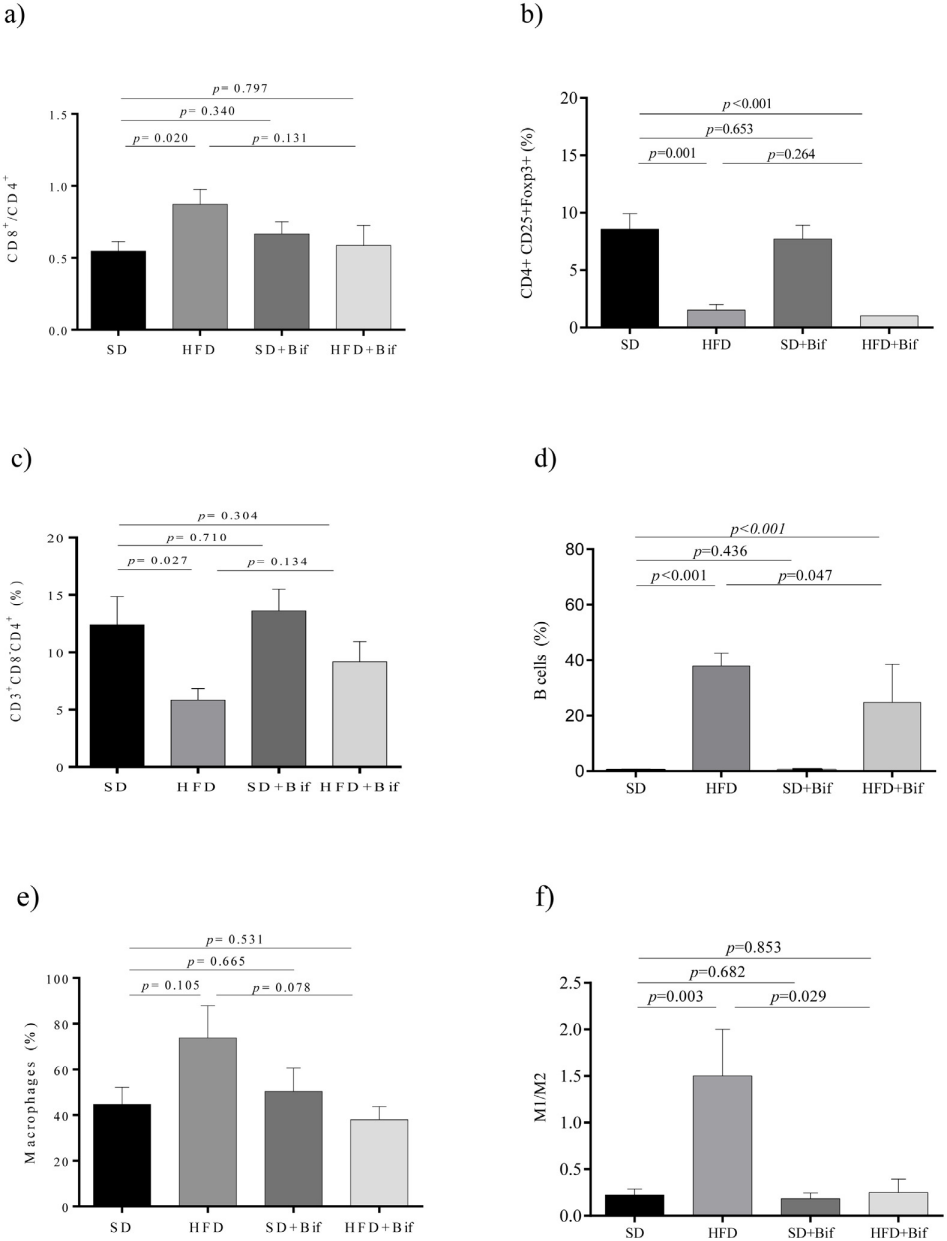
Lymphocyte and macrophage infiltration into epididymal adipose tissue of different mouse groups after 14 weeks of dietary intervention. SD group: control mice receiving a standard diet (SD) plus placebo; HFD group: obese mice receiving a high-fat diet (HFD) plus placebo; SD+Bif group: control mice receiving SD and a daily dose of 1 x 10^9^ CFU *B*. *pseudocatenulatum* CECT 7765; HFD+Bif group: obese mice receiving HFD and a daily dose of 1 x 10^9^ CFU *B*. *pseudocatenulatum* CECT 7765 by gavage during 14 weeks (n = 10). The ratio of CD8^+^/CD4^+^ of total CD3+ (*a*), T regulatory (CD4+CD25+Foxp3+) (*b*), T helper (CD3+CD4+CD8-) (*c*) and B lymphocytes (CD19^+^) (*d*) as well as total macrophages (F4/80+) (*e*) and their subtypes M1 (F4/80+CD11c+CD206-) and M2 (F4/80+CD11c-CD206+) (*f*) were analysed in each mouse group (n = 10 per group) by flow cytometry. Data are expressed as means and standard error (error bars) and statistically significant differences were determined by ANOVA with Bonferroni’s *post hoc* test a *p*<0.050.

Effects of *B*. *pseudocatenulatum* CECT 7765 administration on lymphocyte and macrophage infiltration in adipose tissue from control and obese groups are shown in Fig 2. Obesity induced by HFD increased the CD8+/CD4+ ratio (*p* = 0.020), while reducing the population of CD4+ T cells (*p* = 0.027) and of Foxp3+ Tregs (*p* = 0.001) within the CD3+ CD4+ T cell population in adipose tissue compared to the SD (Fig 2a–2c). *B*. *pseudocatenulatum* CECT 7765 administration did not influence Foxp3+Tregs, but tended to reduce the CD8+/CD4+ ratio (*p* = 0.131) in the obese mouse group. B cells were significantly increased in the HFD group (*p* = 0.001) compared to the SD group and this alteration was ameliorated by the administration of *B*. *pseudocatenulatum* CECT 7765 (*p* = 0.047) (Fig 2d).

Infiltration of F4/80+ macrophages into adipose tissue and specially the M1/M2 ratio were increased by the HFD compared to the SD (*p* = 0.105 and *p* = 0.003, respectively, Fig 2e and 2f), indicating an increase in the proportion of classical activated macrophages (M1) that produce inflammatory cytokines. *B*. *pseudocatenulatum* CECT 7765 administration tended to reduce total macrophages (*p* = 0.078) (Fig 2e) and normalized the M1/M2 ratio in obese mice (*p* = 0.029) (Fig 2f).

### *B*. *pseudocatenulatum* CECT 7765 reduced total macrophage infiltration and the M1 polarization, and increased Tregs in liver of obese mice

**Fig 3:**
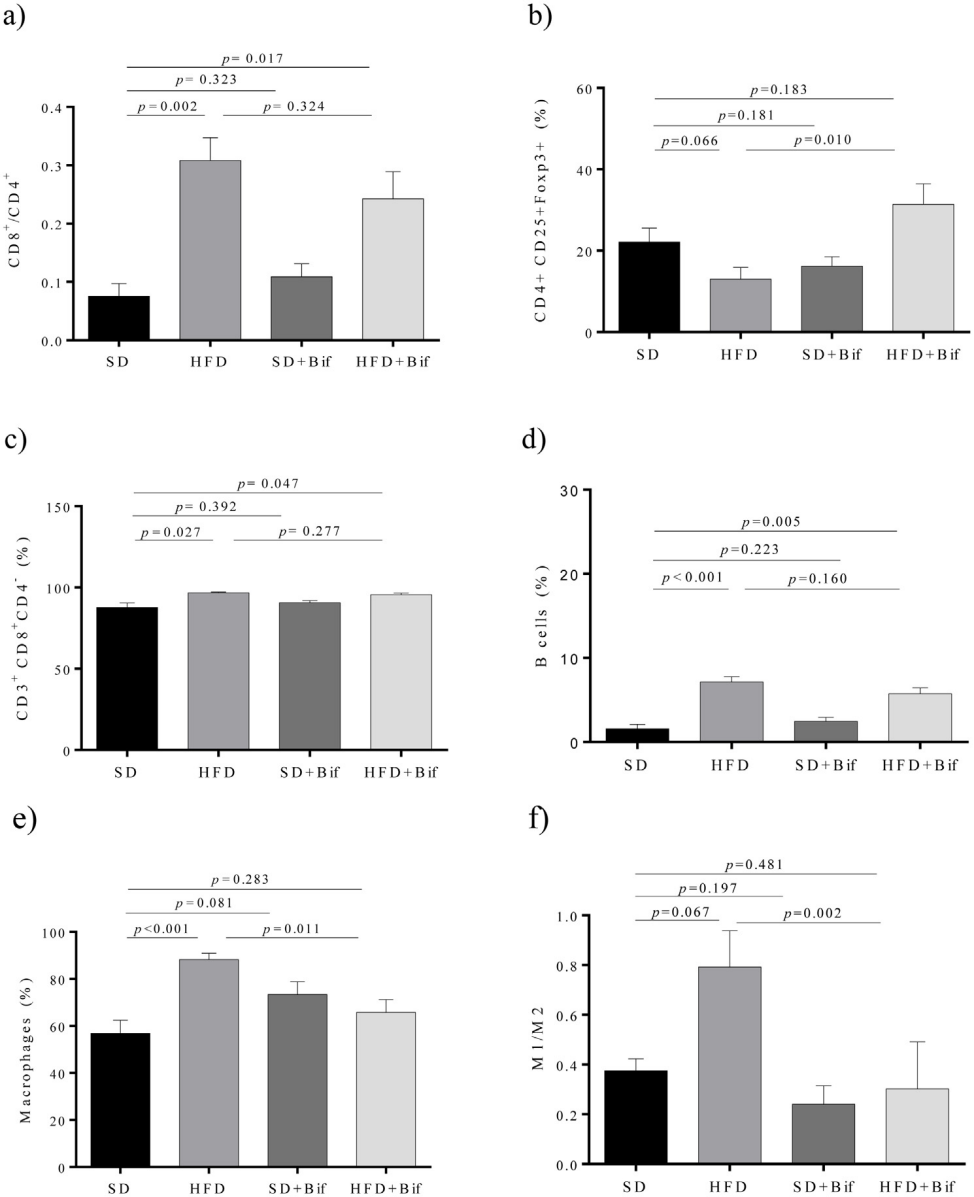
Lymphocyte and macrophage infiltration into the liver of different mouse groups after 14 weeks of dietary intervention. SD group: control mice receiving a standard diet (SD) plus placebo; HFD group: obese mice receiving a high-fat diet (HFD) plus placebo; SD+Bif group: control mice receiving SD and a daily dose of 1 x 10^9^ CFU *B*. *pseudocatenulatum* CECT 7765; HFD+Bif group: obese mice receiving HFD and a daily dose of 1 x 10^9^ CFU *B*. *pseudocatenulatum* CECT 7765 by gavage during 14 weeks (n = 10). The ratio of CD8+/CD4+ of total CD3+ (*a*), T regulatory (CD4+CD25+Foxp3+) (*b*), T cytotoxic (CD3+CD4-CD8+) (*c*) and B lymphocytes (CD19+) (*d*) as well as total of macrophages (F4/80+) (*e*) and their subtypes M1 (F4/80+CD11c+CD206-) and M2 (F4/80+CD11c-CD206+) (*f*) were analyzed in each mouse group (n = 10 per group) by flow cytometry. Data are expressed as means and standard error (error bars) and statistically significant differences were determined by ANOVA with Bonferroni’s *post hoc* test a *p*<0.050.

Effects of *B*. *pseudocatenulatum* CECT 7765 administration on lymphocyte and macrophage infiltration in liver of the SD and the HFD groups are shown in Fig 3. The CD8+/CD4+ ratio (*p* = 0.002; Fig 3a) as well as the CD8+ cell fraction (*p* = 0.027; Fig 3c) were significantly larger in liver of the HFD group than in the SD group mice. *B*. *pseudocatenulatum* CECT 7765 administration did not exert statistically significant effects (*p* = 0.277) on the cytotoxic T cell subpopulation (CD8+). In contrast, *B*. *pseudocatenulatum* CECT 7765 significantly increased the proportion of Tregs in obese mice (*p* = 0.010), which was reduced by the HFD (*p* = 0.066) (Fig 3b). B cells were significantly higher in liver of the HFD group than in the SD group (*p*<0.001), but *B*. *pseudocatenulatum* CECT 7765 did not normalize B cell levels (*p* = 0.160) (Fig 3d).

In liver, macrophage infiltration was significantly increased by the HFD (*p*<0.001) while reduced by the administration of *B*. *pseudocatenulatum* CECT 7765 relative to the obese mouse group (*p* = 0.011) (Fig 3e). M1/M2 ratio was also increased in the HFD group (*p* = 0.067) but significantly reduced (*p* = 0.002) by the administration of *B*. *pseudocatenulatum* CECT 7765 to obese mice (Fig 3f).

### *B*. *pseudocatenulatum* CECT 7765 reduced B cells and increased Tregs in peripheral blood of obese mice

**Fig 4:**
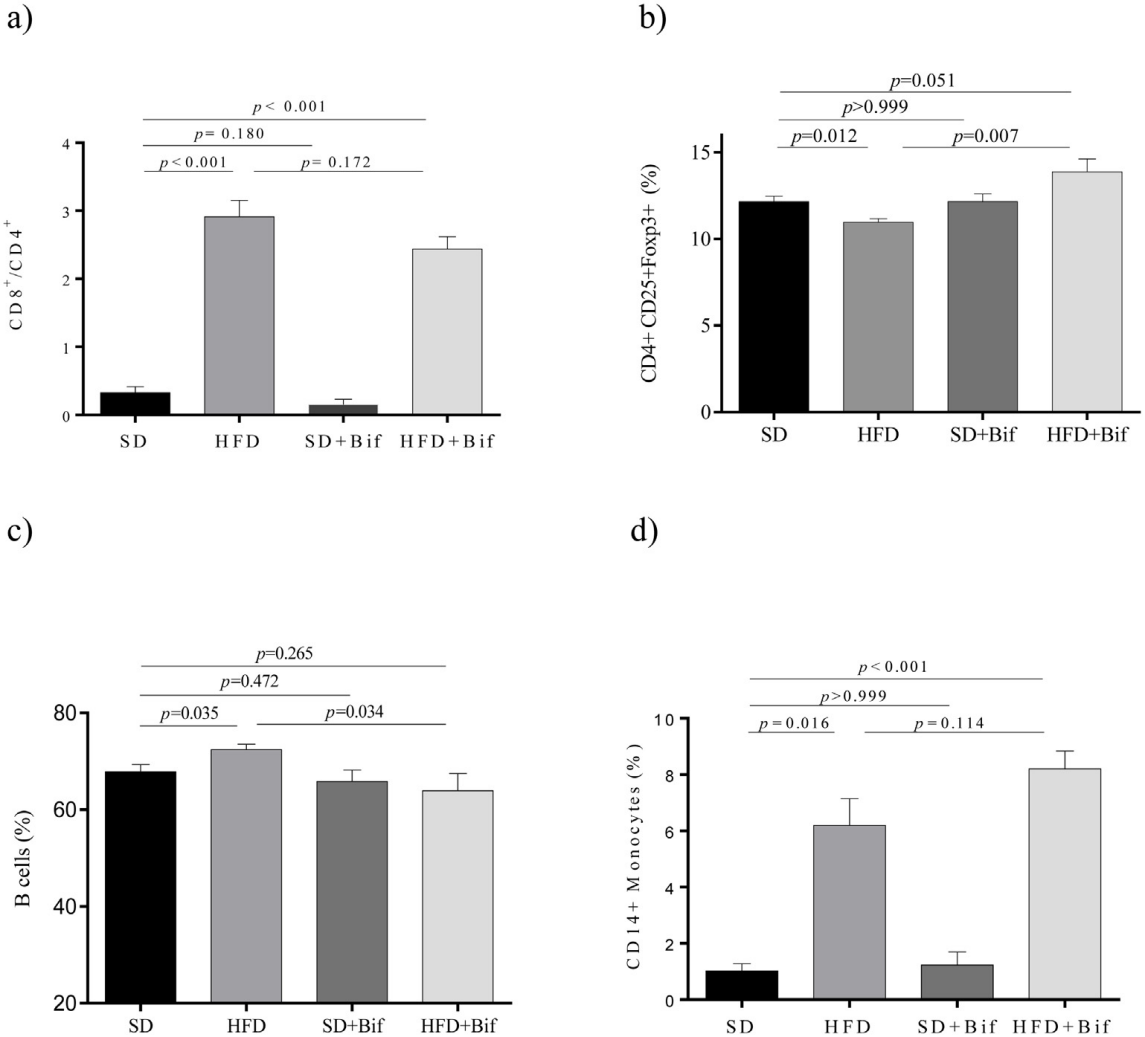
Lymphocyte and monocyte proportions in peripheral blood of different mouse groups after 14 weeks of dietary intervention. SD group: control mice receiving a standard diet (SD) plus placebo; HFD group: obese mice receiving a high-fat diet (HFD) plus placebo; SD+Bif group: control mice receiving SD and a daily dose of 1 x 10^9^ CFU *B*. *pseudocatenulatum* CECT 7765; HFD+Bif group: obese mice receiving HFD and a daily dose of 1 x 10^9^ CFU *B*. *pseudocatenulatum* CECT 7765 by gavage during 14 weeks (n = 10). The ratio of CD8+/CD4+ of total CD3+ (*a*), T regulatory (CD4+CD25+Foxp3+) (*b*) and B lymphocytes (CD19+) (*c*) as well as of monocytes (CD14+) (*d*) were analyzed in each mouse group (n = 10 per group) by flow cytometry. Data are expressed as means and standard error (error bars) and statistically significant differences were determined by ANOVA with Bonferroni’s *post hoc* test a *p*<0.050.

Obesity induced by HFD increased the CD8+/CD4+ ratio (*p*<0.001) in blood compared to SD (Fig 4a), but this was only slightly reduced by *B*. *pseudocatenulatum* CECT 7765 administration (*p* = 0.172). However, *B*. *pseudocatenulatum* CECT 7765 administration significantly increased T regs in obese mice compared to placebo (*p* = 0.007; Fig 4b), which were significantly reduced by the HFD (*p* = 0.012). B cells were also increased in peripheral blood of the HFD group compared to the SD group (*p* = 0.035, Fig 4c), but *B*. *pseudocatenulatum* CECT 7765 significantly reduced their proportions in obese mice (*p* = 0.034; Fig 4c). Monocytes CD14+ were also increased by HFD compared to the SD (*p* = 0.016) but the effects of *B*. *pseudocatenulatum* CECT 7765 were not statistically significant (Fig 4d).

### *B*. *pseudocatenulatum* CECT 7765 reduced B cells and macrophage infiltration and TLR4 expression in small intestine of obese mice

**Fig 5:**
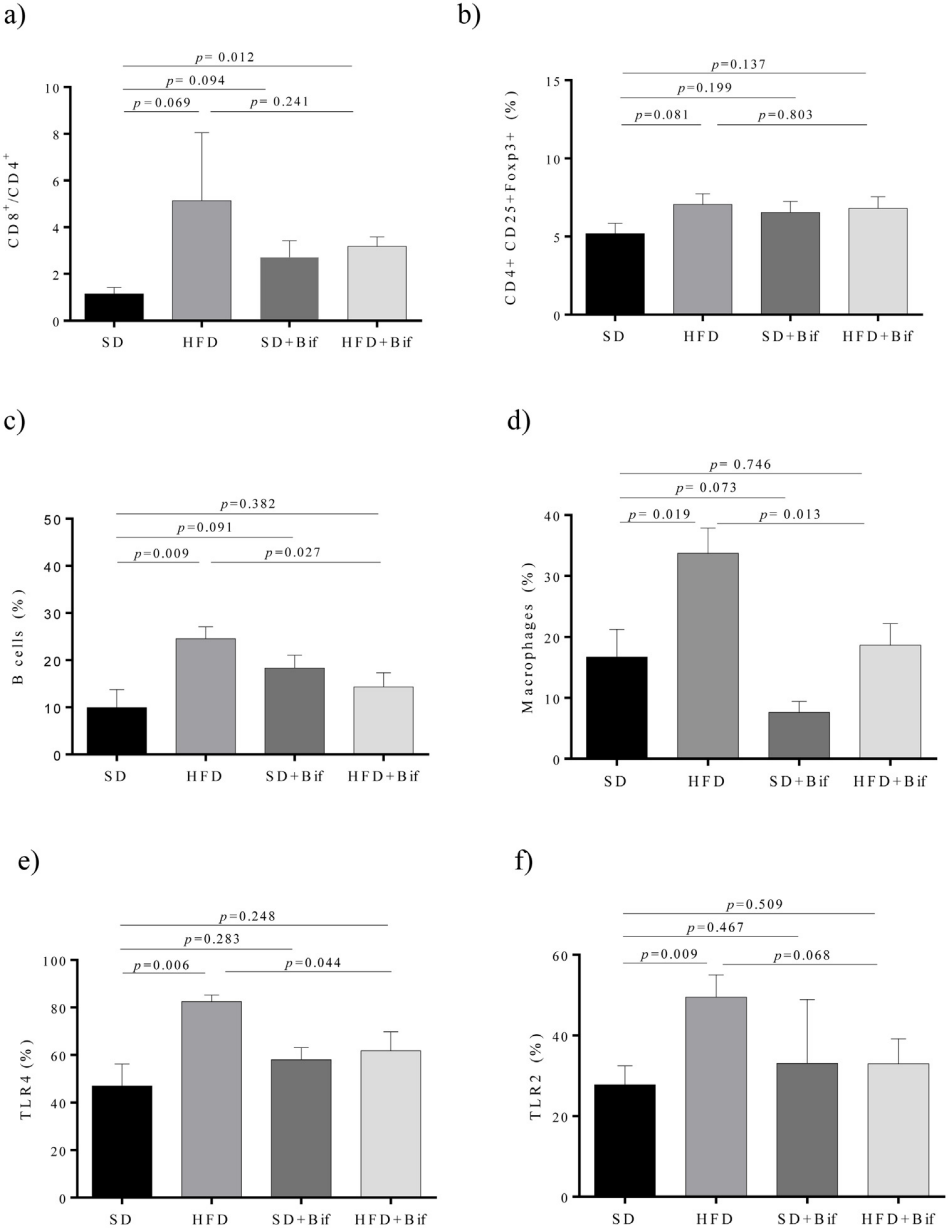
Lymphocyte and macrophage infiltration and TLR4/2 expression in mesenteric lymph nodes (MLNs) from small intestine of different mouse groups after 14 weeks of dietary intervention. SD group: control mice receiving a standard diet (SD) plus placebo; HFD group: obese mice receiving a high-fat diet (HFD) plus placebo; SD+Bif group: control mice receiving SD and a daily dose of 1 x 10^9^ CFU *B*. *pseudocatenulatum* CECT 7765; HFD+Bif group: obese mice receiving HFD and a daily dose of 1 x 10^9^ CFU *B*. *pseudocatenulatum* CECT 7765 by gavage during 14 weeks (n = 10). The ratio of CD8+/CD4+ of total CD3+ (*a*), T regulatory (CD4+CD25+Foxp3+) (*b*) and B lymphocytes (CD19+) (*c*) as well as of total of macrophages (F4/80+) (*d*) and cells expressing TLR4 (CD282+) (*e*) and TLR2 (CD284) (*f*) were determined in each mouse group (n = 10 per group) by flow cytometry. Data are expressed as means and standard error (error bars) and statistically significant differences were determined by ANOVA with Bonferroni’s *post hoc* test a *p*<0.050.

In the small intestine, obesity induced by the HFD slightly increased the CD8+/CD4+ ratio (*p* = 0.069) while this trend was slightly changed by the bifidobacteria administration, but the differences were not statistically significant (Fig 5a). The proportion of B cells was significantly increased in the HFD group compared to the SD group (*p* = 0.009) and *B*. *pseudocatenulatum* CECT 7765 administration led to a significant reduction in this lymphocyte population in obese mice (*p* = 0.027; Fig 5c). Macrophage infiltration was also significantly increased in the HFD group (*p* = 0.019), while significantly reduced by *B*. *pseudocatenulatum* CECT 7765 in obese mice (*p* = 0.013) (Fig 5d). TLR4 and TLR2 expression were also increased significantly in the HFD group (*p* = 0.006 and *p* = 0.009, respectively), while slightly reduced (*p* = 0.044 and *p* = 0.068, respectively) by the administration of *B*. *pseudocatenulatum* CECT 7765 in obese mice (Fig 5e and 5f).

### *B*. *pseudocatenulatum* CECT 7765 reduced cytokine and chemokine concentrations in different tissues and serum of obese mice

#### Adipose tissue

The effects of *B*. *pseudocatenulatum* CECT 7765 administration to the obese and control groups on immune related markers in adipose tissue are shown in Table 2. The HFD significantly induced the production of TNF-α, MCP-1, IP-10, IL-10 and IL-17A in comparison with the SD. However, the administration of *B*. *pseudocatenulatum* CECT 7765 to obese mice significantly reduced production of four of these five cytokines (TNF-α, MCP-1, IP-10, IL-17A; *p* = 0.001–0.016) and the trend was the same for IL-10 (*p* = 0.054). IL-6 was also slightly increased by the HFD (*p* = 0.204), but significantly reduced (*p* = 0.041) by *B*. *pseudocatenulatum* CECT 7765 administration to obese mice.

**Table 2:** Cytokine and chemokine concentrations in tissues and serum from different mouse groups after 14 weeks dietary intervention.

Cytokines (pg/g tissue or ml)	Experimental Groups											
	SD		HFD		SD+Bif		HFD+Bif		*p*-value (HFD vs. SD)	*p*-value (SD+Bif vs. SD)	*p*-value (HFD+Bif vs. HFD)	*p*-value (HFD+ Bif vs.SD)
	Mean	*se*	Mean	*se*	Mean	*se*	Mean	*se*				
**Adipose tissue**												
TNF-α	135.60	19.76	560.50	200.70	143.90	14.95	123.60	3.99	0.014*	0.754	0.025*	0.754
MCP-1	936.30	99.33	4177	732.26	915.80	128.30	1766	205.70	0.001*	0.899	0.005*	0.899
IP-10	56.49	9.83	282.03	53.82	105.60	10.12	131.30	17.12	0.003*	0.006*	0.030*	0.006*
IL-18	512.20	53.37	710.60	80.53	561.5	54.13	563.50	80.68	0.074	0.533	0.221	0.582
IFN-γ	141.30	23.03	263.20	78.78	155.00	17.61	138.51	10.05	0.157	0.635	0.161	0.635
IL-4	75.53	4.91	143.90	32.22	86.48	10.08	79.26	7.70	0.063	0.299	0.081	0.299
IL-6	139.40	10.03	195.50	21.04	157.40	16.37	137.70	14.63	0.204	0.341	0.041*	0.341
IL-10	118.00	24.76	214.00	23.77	124.50	18.09	150.10	18.44	0.016*	0.865	0.054	0.212
IL-1β	535.20	52.10	497.00	47.65	580.60	86.64	572.60	87.28	0.599	0.651	0.446	0.651
IL-17A	222.20	36.14	621.00	33.92	345.90	60.58	352.90	25.14	0.002*	0.046*	0.005*	0.073
**Liver**												
TNF-α	118.20	8.99	328.09	43.69	110.92	11.21	159.72	31.36	<0.001*	0.624	0.011*	0.232
MCP-1	5551.19	331.39	5919.83	381.22	4447.38	218.28	5458.95	414.20	0.542	0.015*	0.476	0.059
IP-10	*n*.*d*		*n*.*d*		*n*.*d*		*n*.*d*					
IL-18	4638.13	812.70	16051.94	1923	5173.91	1760	11032.21	2634	<0.001*	0.776	0.172	0.019*
IFN-γ	70.38	5.44	321.23	68.75	52.56	6.06	102.19	32.80	0.007*	0.054	0.049*	0.264
IL-4	*n*.*d*		*n*.*d*		*n*.*d*		*n*.*d*					
IL-6	1974.81	223.50	1653.37	255.11	1184.95	238.84	1132.77	208.07	0.485	0.233	0.286	0.042*
IL-10	1360.00	25.70	3000.00	271.13	1369.00	94.49	1249.00	103.01	0.064	0.964	0.056	0.964
IL-1β	130.11	16.08	498.23	108.30	85.34	24.08	74.89	3.03	0.021*	0.189	0.026*	0.011*
IL-17A	252.00	27.41	328.10	30.37	272.00	11.22	248.70	12.51	0.093	0.514	0.020*	0.863
**Serum**												
TNF-α	20.19	4.55	138.50	14.84	94.25	67.16	60.83	28.58	<0.001*	0.072	0.050*	0.069
MCP-1	303.05	0.07	569.84	116.49	686.33	1.15	637.64	187.38	0.084	<0.001*	0.687	<0.001*
IP-10	*n*.*d*		*n*.*d*		*n*.*d*		*n*.*d*					
IL-18	476.03	61.97	1057.66	294.11	736.57	196.40	678.70	173.01	0.013*	0.051	0.076	0.072
IFN-γ	32.36	9.87	52.36	24.80	38.44	17.12	37.42	12.97	0.058	0.447	0.191	0.661
IL-4	*n*.*d*		*n*.*d*		*n*.*d*		*n*.*d*					
IL-6	52.64	3.73	56.05	8.23	60.14	16.10	58.53	19.39	0.621	0.587	0.818	0.529
IL-10	*n*.*d*		*n*.*d*		*n*.*d*		*n*.*d*					
IL-1β	1094	132.94	995.09	175.40	855.64	50.94	989.53	162.50	0.553	0.058	0.969	0.519
IL-17A	18.71	1.86	26.41	1.94	22.70	3.54	18.04	1.99	0.011*	0.320	0.009*	0.773
**Small intestine**												
TNF-α	80.16	4.03	135.00	20.30	85.44	7.36	87.16	10.63	0.012*	0.576	0.021*	0.815
MCP-1	994.40	75.86	3349	281.00	1004	124.30	1708	185.80	<0.001*	0.946	<0.001*	0.005*
IP-10	125.90	28.80	328.60	59.63	121.60	17.76	171.60	35.13	0.017*	0.898	0.042*	0.151
IL-18	462.10	76.25	1146	135.70	708.50	99.50	708.70	123.90	<0.001*	0.065	0.028*	0.145
IFN-γ	124.60	11.96	159.50	12.62	129.50	11.30	131.50	12.63	0.063	0.770	0.139	0.939
IL-4	66.81	4.96	110.10	16.10	72.14	9.86	69.95	6.94	0.025*	0.613	0.035*	0.764
IL-6	127.11	11.32	170.60	15.01	132.10	11.44	130.50	8.86	0.032*	0.769	0.035*	0.966
Il-10	133.10	21.79	209.30	18.66	163.90	24.71	162.50	16.57	0.016*	0.362	0.080	0.200
IL-1β	231.90	27.89	252.41	32.29	224.50	48.49	237.70	55.85	0.634	0.889	0.835	0.921
IL-17A	36.03	10.40	104.60	19.40	47.66	6.05	35.76	7.96	0.013*	0.501	0.012*	0.976

SD group: control mice receiving a standard diet (SD) plus placebo; HFD group: obese mice receiving a high-fat diet (HFD) plus placebo; SD+Bif group: control mice receiving SD and a daily dose of 1 x10^9^ CFU *B*. *pseudocatenulatum* CECT 7765; HFD+Bif group: obese mice receiving HFD and a daily dose of 1 x10^9^ CFU *B*. *pseudocatenulatum* CECT 7765 by gavage during 14 weeks. Data are expressed as mean and standard error of the mean (se)(n = 10 per group).

*Significant differences were established by ANOVA and *post hoc* Bonferroni’s test at p≤0.050. Symbol *n*.*d*. indicates non-detectable values.

#### Liver

The HFD significantly induced (*p*<0.001–0.021) the production of TNF-α, IL-18, IFN-γ and IL-1β and also slightly increased IL-10 (*p* = 0.064) and IL-17A (*p* = 0.093) compared to the SD in liver (Table 2). Therefore, the only inflammatory cytokine commonly involved in both adipose tissue and liver inflammation that changed significantly was TNF-α, which increased by 313% and 178%, respectively. *B*. *pseudocatenulatum* CECT 7765 reduced the concentrations of TNF-α, IFN-γ, IL-1β and IL-17A compared to obese mice fed placebo (*p* = 0.011–0.049). In liver, the administration of *B*. *pseudocatenulatum* CECT 7765 to control mice also significantly reduced MCP-1 (*p* = 0.015) and the same trend was detected for IFN-γ (*p* = 0.054), which seemed to be an effect of the bifidobacteria independent of diet (Table 2).

#### Serum

The most significant alterations of immune markers associated with HFD-induced obesity in serum were those of TNF-α, IL-18 and IL-17A, whose concentrations significantly increased (*p*<0.001–0.013; Table 2). The same trend was detected for MCP-1 and IFN-γ, but the differences did not reach statistical significance (*p* = 0.084 and *p* = 0.058, respectively; Table 2). *B*. *pseudocatenulatum* CECT 7765 significantly reduced TNF-α (*p* = 0.050) and IL-17A (*p* = 0.009) and the same trend was detected for IL-18 (*p* = 0.076) in obese mice.

#### Small intestine

The HFD significantly induced the production of most of the cytokines analysed in the gut including TNF-α, MCP-1, IP-10, IL-18, IL-4, IL-6, IL-10 and IL-17A and the increases were also almost significant for IFN-γ (*p* = 0.063; Table 2). *B*. *pseudocatenulatum* CECT 7765 administration significantly reduced all the immune markers induced by the HFD except for IL-10. *B*. *pseudocatenulatum* CECT 7765 administration to control mice also tended to reduce IL-18 production (*p* = 0.065), which seemed to be an effect of the bifidobacteria independent of diet (Table 2).

### HFD-induced metabolic endotoxemia is reduced by *B*. *pseudocatenulatum* CECT 7765

**Fig 6:**
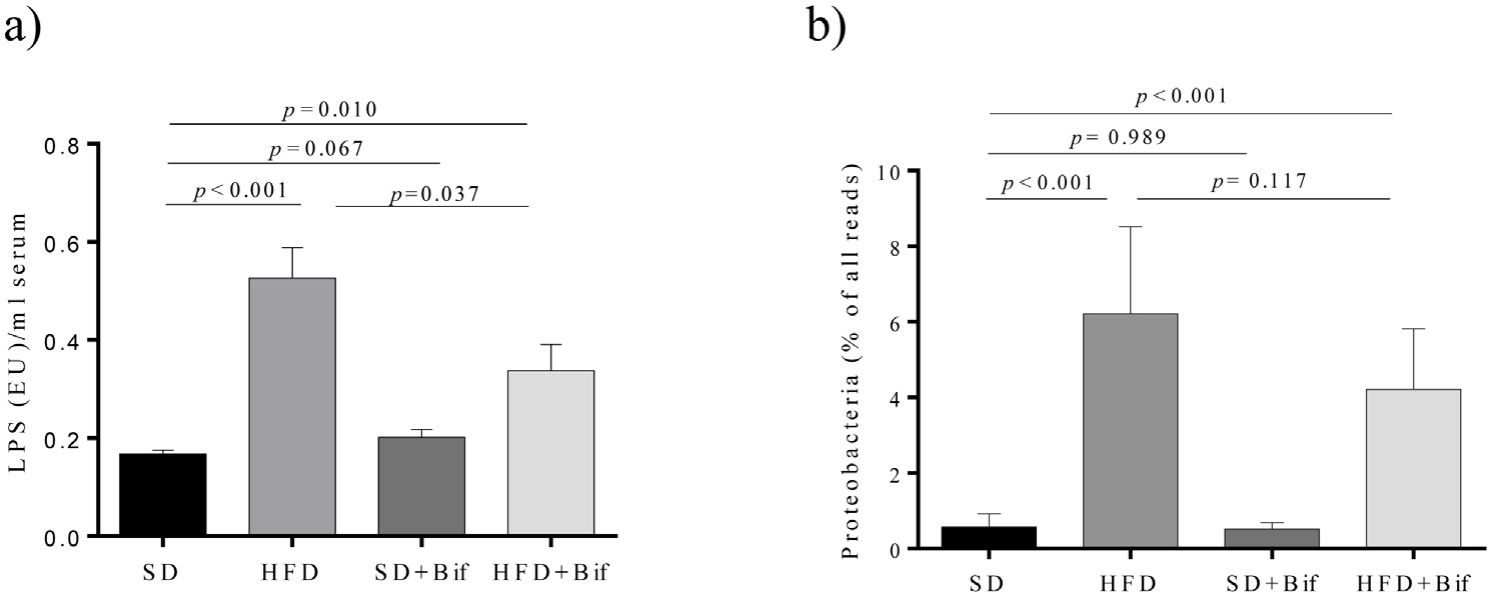
Lipopolysaccharide (LPS) concentration in serum(a) and Proteobacteria abundance in faeces of different mouse groups after 14 weeks of dietary intervention (b). SD group: control mice receiving a standard diet (SD) plus placebo; HFD group: obese mice receiving a high-fat diet (HFD) plus placebo; SD+Bif group: control mice receiving SD and a daily dose of 1 x 10^9^ CFU *B*. *pseudocatenulatum* CECT 7765; HFD+Bif group: obese mice receiving HFD and a daily dose of 1 x 10^9^ CFU *B*. *pseudocatenulatum* CECT 7765 by gavage during 14 weeks. LPS concentrations are expressed as means and standard errors (error bars) of data from each mouse group (n = 8 per group) and statistically significant differences were determined by ANOVA with Bonferroni’s *post hoc* test at *p* <0.050 (a). Proteobacteria abundances as relative fractions of all 454 reads in each sample are represented as means and standard errors (error bars) of data from each mouse group (n = 8) and statistically significant differences were determined using a permutation test at a p < 0.050 (b).

Plasma endotoxin (LPS) concentrations were higher in the HFD group than in the SD group mice (Fig 6a); however, the administration of *B*. *pseudocatenulatum* CECT 7765 reduced metabolic endotoxemia in obese mice (*p* = 0.037). No effects on control mice were detected. Proteobacteria are among the best known sources for LPS and were almost absent in the SD group and the SD+Bif group, while significantly increased in HFD groups (Fig 6b). The administration of *B*. *pseudocatenulatum* CECT 7765 to obese mice tended to reduce Proteobacteria levels (p = 0.117) parallel to LPS reduction in serum (Fig 6).

### *B*. *pseudocatenulatum* CECT 7765 administration partially restored the HFD-induced alterations in gut microbiota structure

**Fig 7:**
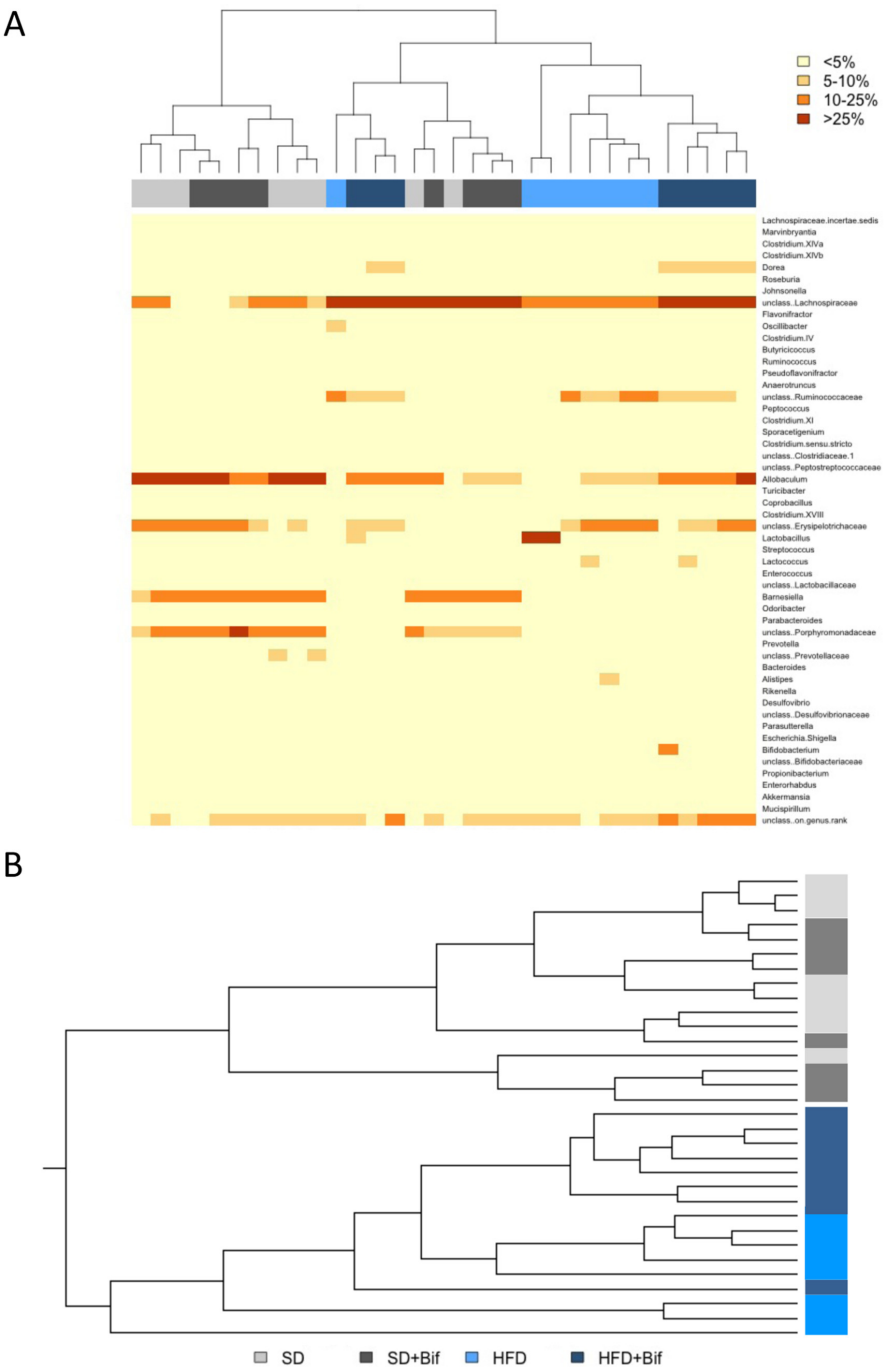
Cluster analysis of the fecal microbiota composition determined by pyrosequencing in different mouse groups after 14 weeks of dietary intervention. Mouse group affiliation is indicated in light-grey and dark-grey for the SD group and the SD+Bif group and light-blue and dark-blue for the HFD group and the HFD+Bif group mice, respectively. A: Heatmap of Bray-Curtis distances of 52 genera with abundances of at least 0.1% in at least one sample. Abundances are categorized in four classes: below 5%, 5 to 10%, 10 to 25% and above 25% of the sequence reads. B: Cluster dendrogram of the weighted UniFrac analysis of 1254 phylotypes with more than 10 reads in all samples of the four groups.

**Fig 8:**
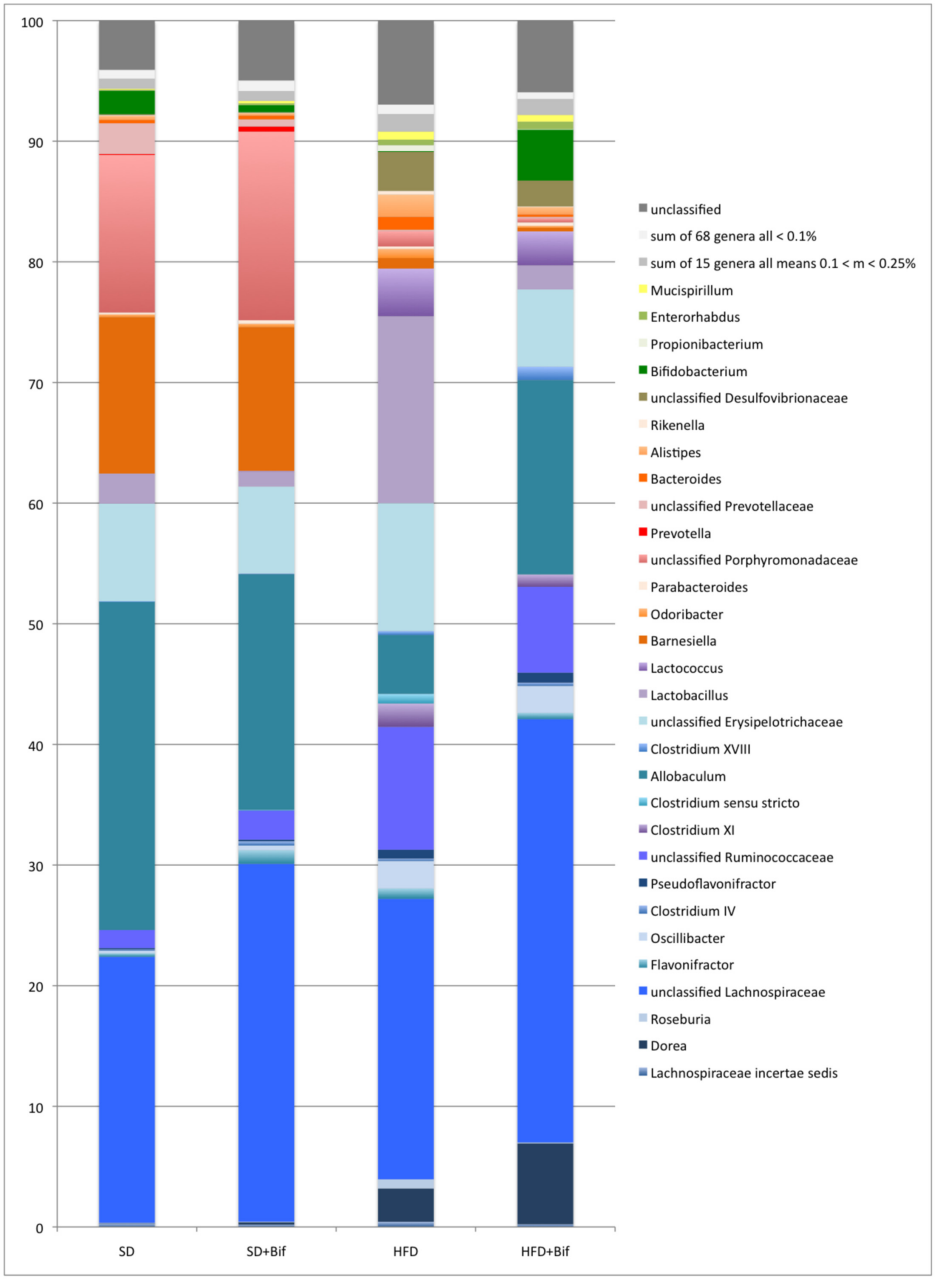
Microbiota composition as determined by pyrosequencing with the RDP multiclassifier in faeces from different mouse groups after 14 weeks of dietary intervention. Data are expressed as mean percentage values from each mouse group (n = 8 per group). Phylum affiliation of the genera is indicated by blue tones for Firmicutes, orange-red for Bacteroidetes, brown for Proteobacteria, green for Actinobacteria and yellow for Deferribacteres. Genera with abundances below 0.1% and between 0.1 and 0.25% are grouped. Reads that had confidence values below 80% at family level are shown as "unclassified".

Compositional analysis of gut microbiota structure by pyrosequencing (7,844 [mean], 4,192 [se] reads per sample on average) revealed that HFD is a major factor influencing gut microbiota structure. The weighted UniFrac clustering analysis principally separated the SD and the HFD mouse group’s microbiota (Fig 7B). All samples from the HFD+Bif group except one clustered together whereas among those from the control groups (fed a SD), the ones with and without *B*. *pseudocatenulatum* administration, were intermingled. Bray-Curtis clustering analysis also showed only a partial grouping of the samples according to the four experimental mouse groups (Fig 7A). Taxonomic assignment with the RDP multiclassifier showed specific profiles for each of the four mouse groups, but also revealed general similarities between the two groups fed a SD, supplemented (SD+Bif group) or not with the bifidobacteria (SD group), and between the two groups fed the HFD (HFD group and HFD+Bif group) (Fig 8, Table in S3 File).

On phylum level, Firmicutes dominated the microbiota of the SD group and the SD+Bif group representing 65 and 66% of all reads, respectively, followed by Bacteroidetes, which represented 31% (Table in S3 File). In the microbiota of both HFD-fed mouse groups, Firmicutes were significantly increased (*p*<0.001 for both comparisons, SD *versus* HFD group and SD+Bif *versus* HFD+Bif group), while Bacteroidetes were reduced (*p*<0.001 for both comparisons). Additionally, HFD feeding increased the proportion of Proteobacteria compared to SD feeding (*p*<0.001 for both comparisons).

On family level, Porphyromonadaceae showed significantly lower abundance in both HFD-fed mouse groups (HFD and HFD+Bif) than in both SD-fed mouse groups (SD and SD+Bif) (*p*<0.001 for all comparisons). In contrast, *Ruminococcaceae* were more abundant in both HFD-fed mouse groups than in both SD-fed mouse groups (*p*<0.001–0.002). Desulfovibrionaceae, a major group of Proteobacteria, was also more abundant in HFD-fed mice than in SD-fed mice (*p*<0.001; Table in S3 File).

On genus level (Fig 7, Table in S3 File, *Allobaculum*, *Barnesiella* and unclassified Porphyromonadaceae were significantly less abundant in both HFD-fed mouse groups than in both SD-fed mice (for all comparisons *p*<0.001) (Table 3). In contrast, *Lactobacillus*, unclassified Ruminococcaceae, *Lactococcus*, unclassified Desulfovibrionaceae, *Oscillibacter*, *Clostridium* XI, and *Alistipes* were more abundant in the HFD-fed group than in the SD-fed group (*p*<0.001–0.044).

**Table 3:** Average abundances of bacterial taxa detected in the microbiota of different mouse groups after 14 weeks dietary intervention.

Taxa/Genera	Relative abundance of taxa in all 454 reads (%)								*p*-values in different experimental mouse groups			
	SD		HFD		SD+Bif		HFD+Bif		HFD vs.SD	SD vs. SD+Bif	HFD vs. HFD+Bif	HFD vs. HFD+Bif
	Mean	*se*	Mean	*se*	Mean	*se*	Mean	*se*				
*Dorea*	0.07	0.05	2.76	0.68	0.22	0.14	6.71	2.05	<0.001*	0.111	<0.001*	<0.001*
uncl. Lachnospiraceae	22.05	19.64	23.26	9.30	29.66	23.61	35.1	7.00	0.892	0.495	0.027*	0.133
*Flavonifractor*	0.30	0.22	0.87	0.75	1.14	1.39	0.52	0.19	0.049*	0.127	0.227	0.056
*Oscillibacter*	0.20	0.12	2.27	1.41	0.38	0.23	2.23	0.80	<0.001*	0.048* 0.048*00.048*0.048*	0.886	<0.001*
uncl. Ruminococcaceae	1.50	0.74	10.19	5.65	2.45	1.60	7.12	1.74	<0.001*	0.152	0.151	<0.001*
*Clostridium* XI	0.00	0.00	1.94	1.41	0.00	0.00	1.03	0.19	<0.001*	n.a.	0.074	<0.001*
*Allobaculum*	27.24	12.06	4.89	3.17	19.54	12.78	16.11	6.29	<0.001*	0.230	<0.001*	0.054
*Clostridium* XVIII	0.02	0.00	0.33	0.12	0.06	0.05	1.11	0.65	<0.001*	0.067	<0.001*	< 0.001*
uncl. Erysipelotrichaceae	8.09	6.14	10.59	8.75	7.20	6.66	6.39	2.27	0.507	0.784	0.284	0.648
*Lactobacillus*	2.49	1.11	15.49	22.63	1.29	1.04	2.00	2.48	0.044*	0.044* 0.044*	0.047*	0.568
*Lactococcus*	0.00	0.00	3.95	1.19	0.02	0.00	2.81	1.66	<0.001*	n.a.	0.206	<0.001*
*Barnesiella*	12.97	3.54	0.87	0.72	11.92	1.77	0.30	0.25	<0.001*	0.470	0.030*	<0.001*
uncl. Porphyromonadaceae	13.07	3.29	1.28	0.78	15.63	9.97	0.35	0.23	<0.001*	0.506	<0.001*	<0.001*
uncl. Prevotellaceae	2.54	2.00	0.11	0.16	0.59	0.26	0.11	0.00	<0.001*	<0.001*<0.001*	0.056	<0.001*
*Bacteroides*	0.28	0.23	1.08	1.17	0.31	0.13	0.19	1.44	<0.001*	0.793	<0.001*	0.363
*Alistipes*	0.36	0.12	1.84	1.78	0.20	0.13	0.63	0.68	0.007*	0.028*	0.066	0.475
uncl. Desulfovibrionaceae	0.09	0.09	3.26	1.28	0.04	0.04	2.15	0.79	<0.001*	0.331	0.082	< 0.001*
*Bifidobacterium*	1.95	1.58	0.03	0.04	0.58	0.33	4.26	5.48	<0.001*	0.030*	<0.001*	0.213

Taxa included are those with abundances of at least 1% of the reads in the microbiota in at least one mouse group. SD group: control mice receiving a standard diet (SD) plus placebo; HFD group: obese mice receiving a high-fat diet (HFD) plus placebo; SD+Bif group: control mice receiving SD and a daily dose of 1 x 10^9^ CFU *B*. *pseudocatenulatum* CECT 7765; HFD+Bif group: obese mice receiving HFD and a daily dose of 1 x 10^9^ CFU *B*. *pseudocatenulatum* CECT 7765 by gavage during 14 weeks. (n = 8 per mouse group).

* Statistically significant differences were determined using a permutation test at a *p* value ≤ 0.05. n.a.: not applicable, uncl: unclassified.

The HFD also has an important adverse impact on the genus *Bifidobacterium*, which was significantly less abundant in the HFD than in the SD group (*p*<0.001) (Table 3). However, *B*. *pseudocatenulatum* CECT7765 administration partially restored this and other alterations induced by the HFD in the gut ecosystem. Intervention with the bifidobacterial strain significantly increased *Bifidobacterium* proportions (*p*<0.001) in obese mice. Searches for the closest reference sequence via BlastN showed that in SD group mice members or relatives of four different species were present with a dominance of *B*. *pseudolongum* (98.3%), while *B*. *pseudocatenulatum* was not detected (Fig in S4 File.). In the SD+Bif group, *B*. *pseudolongum* still prevailed among the five species detected, representing 91% of all *Bifidobacterium* reads, whereas 5% of the reads belonged to *B*. *pseudocatenulatum*. In the HFD+Bif group, 97% of the *Bifidobacterium* population consisted of *B*. *pseudocatenulatum* demonstrating that the administered bacteria colonized the gut of obese mice despite the general adverse effect of the HFD on *Bifidobacterium* levels. On the contrary, in the SD+Bif group *B*. *pseudocatenulatum* was not so dominant, probably due to the presence of autochthonous *B*. *pseudolongum*, which presumably contributed to gut colonization resistance against allochthonous bifidobacteria orally administered. Accordingly, real-time quantification of rRNA gene copy numbers also revealed a significant reduction of *Bifidobacterium* spp. in the HFD group compared to the SD group (*p* = 0.001) (data not shown). In obese mice, *B*. *pseudocatenulatum* CECT 7765 administration increased the gene copy numbers of *Bifidobacterium* spp. (*p*<0.001) contributing to gut ecosystem restoration. In control mice, *B*. *pseudocatenulatum* CECT 7765 also increased total bifidobacterial gene copy numbers (*p* = 0.017).

Administration of *B*. *pseudocatenulatum* CECT7765 to the obese mice also resulted in an increase of the amounts of *Allobaculum* (*p*<0.001), unclassified Lachnospiraceae (*p* = 0.027) and *Dorea* (*p*<0.001) proportions (Table 3). In parallel, it also reduced the proportions of *Lactobacillus* (*p* = 0.047), uncl. Porphyromonadaceae (*p* = 0.001) and *Bacteroides* (*p*<0.001) in obese mice compared to placebo (Table 3). Other bacterial groups were not significantly restored by the bifidobacterial intervention in obese mice, including unclassified Ruminococcaceae, *Lactococcus*, and unclassified Desulfovibrionaceae, which remained at elevated levels compared to control mice (*p*<0.001 for SD group and HFD+Bif group). Also *Barnesiella* and unclassified Porphyromonadaceae, which were reduced by the HFD, did not increase significantly as a result of the bifidobacterial intervention (*p*<0.001 for SD group and HFD+Bif group). In all four mouse groups, approximately half of the reads belonged to phylotypes that could not be classified reliably at genus level (at 80% confidence), especially among the Firmicutes and also among Bacteroidetes.

BlastN comparisons against a 16S rRNA gene database were also performed to acquire additional information on the phylogenetic affiliation of the reads detected. However, sequence similarities of the identified phylotypes to reference sequences of bacterial species were relatively low (91.3±4.6% on average) (data not shown). Only 11% of the reads shared a similarity of 97% or higher with its closest reference, a value considered as a delimitation to define species affiliation among bacteria.

Rarefaction curves did not differ significantly between the four groups, but SD+Bif group showing the highest values and the HFD and HFD+Bif groups the lowest values (data not shown). Comparison of species richness, Shannon and Simpson indices for community diversity did not show significant differences between the four groups at any of the three similarity cut-offs (100, 97 and 90%) tested (data not shown).

## Discussion

### *B*. *pseudocatenulatum* CECT 7765 reduces obesity-associated adipose tissue inflammation by reducing B cells and components of innate immunity

Our study has demonstrated for the first time that a *Bifidobacterium* strain (*B*. *pseudocatenulatum* CECT 7765) reduces B lymphocyte infiltration, thereby contributing to reducing adipose tissue inflammation in obese mice. This lymphocyte population was significantly increased in HFD induced obesity in mice as described previously [27,28]. B lymphocytes are reported to be one of the first immune cells recruited into adipose tissue shortly after initiation of a HFD in mice, and they adversely influence glucose metabolism via activation of pro-inflammatory T cells and macrophages [28]. The involvement of B cells in obesity-associated metabolic dysfunction is also supported by the increased insulin sensitivity found in B-cell-deficient mice on HFD compared with wild-type controls [28]. Adipose tissue-associated B cells can contribute *per se* to production of inflammatory cytokines, such as IL-8 and IFN-γ [29]. Adipose tissue-associated B cells can also induce MHC-dependent release of pro-inflammatory cytokines (e.g. IFN-γ) from resident T lymphocytes and induce macrophage polarization into the M1 phenotype, thereby contributing to the inflammatory cascade [4,28]. Increased CD8+ T cell infiltration and accumulation have also been described as an additional critical event driving adipose tissue inflammation with production of pro-inflammatory cytokines (e.g. IFN-γ) that contribute to macrophage activation [6]. Increased CD8+ numbers were usually paralleled by decreases in CD4+ numbers and, therefore, increased CD8+/CD4+ ratio in the HFD group [6,30]. An increased CD8+/CD4+ ratio has also been detected in adipose tissue of our obese mouse model, but the effects of our bifidobacteria on T lymphocyte proportions (CD8+/CD4+) were modest and non-significant. In our study, the proportion of Treg cells was drastically reduced in obese adipose tissue paralleled to increases in B cells, as described by other authors in obesity and insulin resistance animal models [5,6]. In contrast, Treg cells are described to be enriched in the abdominal fat of normal mice and to have an unusual high state of activation, presumably contributing to down-regulate the inflammatory state and prevent insulin resistance [5,30]. A role for Tregs in glucose metabolism is supported by studies showing that diphtheria toxin-induced depletion of Tregs cells was accompanied by decreases in insulin-stimulated insulin-receptor (IR) tyrosine phosphorylation in epidydimal fat and liver [5]. However, no significant effects of *B*. *pseudocatenulatum* CECT 7765 intervention were detected in Tregs in this tissue. In contrast, *Akkermansia muciniphila* Muc^T^ has been shown to reduce adipose tissue inflammation by increasing the generation of Tregs, whereas effects on the CD4+/CD8+ ratio were not detected and effects on B cells were not evaluated [30].

Our study also shows that *B*. *pseudocatenulatum* CECT 7765 effectively reduces adipose tissue inflammation by reducing innate immune mediators (macrophages and related cytokines). In particular, *B*. *pseudocatenulatum* CECT 7765 reduced the ratio of pro-inflammatory (M1) to anti-inflammatory (M2) macrophages. Accordingly, the bifidobacteria also reduced the HFD-induced cytokines mainly produced by pro-inflammatory macrophages (TNF-α, IL-6) or acting as chemoattractants of macrophages (MCP-1 and IP-10). However, the effects of *A*. *muciniphila* Muc^T^ in reducing macrophage (M1/M2) polarization have not been consistent across studies [30,31]. *A*. *muciniphila* Muc^T^ administration also led to down-regulate IL-6 and IL-1β gene expression, but did not influence the expression of other key cytokines in adipose tissue inflammation such as TNF-α or chemokines [30].

### *B*. *pseudocatenulatum* CECT 7765 reduces obesity-associated liver inflammation by increasing Tregs and reducing macrophages and cytokine concentrations

Our study has also demonstrated for the first time that the administration of an indigenous human gut bacterium (*B*. *pseudocatenulatum* CECT 7765) reduced hepatic inflammation associated with HFD-induced obesity in mice by shifting cellular components of adaptive immunity and, specifically, by increasing the Tregs population. *B*. *pseudocatenulatum* CECT 7765 also tended to reduce B cells and the CD8+/CD4+ ratio, whose increase is associated with serious liver alterations (e.g. fibrosis), but these latter changes were not statistically significant. However, the fact that the bifidobacteria reduced the IFN-γ concentration in adipose tissue suggests that it also ameliorated lymphocyte infiltration responsible for the production of this cytokine. The administration of *B*. *pseudocatenulatum* CECT 7765 to control mice also reduced IFN-γ (*p* = 0.054) suggesting that effects on this cytokine are mediated by the bacteria regardless the diet. IFN-γ is considered the hallmark pro-inflammatory cytokine produced by T lymphocytes that also enhance the production of other inflammatory cytokines (e.g. TNF-α) through macrophage activation in the adipose tissue, which can also be the case for liver. Unlike our results, the probiotic mixture VSl#3 was shown to exert a positive effect on insulin resistance and steatosis in liver of mice under HFD by restoration of an associated NKT cell depletion [32].

*B*. *pseudocatenulatum* CECT 7765 administration significantly reduced both total macrophage infiltration and the M1/M2 ratio in the liver of obese mice, whose increases are characteristic of diet-induced obesity and contribute to insulin resistance [33,34]. Likewise, our bifidobacterial strain also reduced the HFD-induced increase of cytokines of innate immunity (TNF-α and IL-1β) parallel to improvements in metabolic markers and functions, including serum lipid concentrations, glucose tolerance and insulin sensitivity. In this context, the probiotic product VSL#3 administered to HFD-fed rats was also shown to reduce liver TNF-α levels and expression of iNOS and COX-2 and increased PPARα expression suggesting reductions in oxidative and inflammatory processes [33].

MCP-1 is the dominant signal controlling the recruitment of macrophages in liver and it is reported to derive from circulating monocytes [35]. However, in our study this chemokine was not significantly increased in the liver of the HFD group, suggesting that regulation of MCP-1 levels in other tissues such as the small intestine and the adipose tissue influences the recruitment of macrophages in the liver via communication through the gut-peripheral tissue axis. In contrast, liver macrophages seemed to be the main contributors to the locally increased IL-1β concentrations since their levels were not increased in gut, serum, or adipose tissue of HFD-mice.

### *B*. *pseudocatenulatum* CECT 7765 reduces obesity-associated inflammation by reducing metabolic endotoxemia and pro-inflammatory cytokine concentrations and by restoring the balance between Tregs and B lymphocytes in systemic circulation

The anti-inflammatory effects of the *Bifidobacterium* strain detected in the adipose tissue and liver could be partly related to its ability to reduce LPS translocation from the gut to the blood stream, thus reducing metabolic endotoxemia and systemic inflammation in obese mice. This hypothesis is supported by the ability of the *Bifidobacterium* strain tested to reduce serum cytokines primarily produced by activated innate immune cells in response to LPS stimulation, such as TNF-α and IL-18. In obese mice, LPS could also reach peripheral tissues and activate inflammatory pathways locally via TLR4 signalling which lead to increased expression of inducible nitric oxide synthase (iNOS) that reacts with cysteine residues interfering with the insulin signalling pathway and leading to insulin resistance in the liver and adipose tissue. IL-18 also contributes to inducing cell-mediated immunity and can stimulate certain T cells to release IFN-γ that in turn activates monocyte/macrophages, which can generate a positive feed-back loop inflammatory process.

*B*. *pseudocatenulatum* CECT 7765 also seems to reduce systemic inflammation by the activation of immunoregulatory mechanisms since its administration to obese mice increased Tregs and reduced B cells and IL-17A in peripheral blood/serum, which suggests an attenuation of the Th17 response described in obese mice. B cells have been reported to primarily contribute to inflammation and insulin resistance via induction of an inflammatory T cell ratio (reduced Treg cells and increased Th1/Th17 cells) and production of pro-inflammatory cytokines (e.g. IL-17A) in obese mice and also in obese human subjects [7,36]. A recent study also reported that obese mice fed a Western chow diet had higher serum IL-17 concentrations than control mice and that *L*. *reuteri* ATCC 6475 administration reduced the levels of this cytokine, presumably by restoring Foxp3+ Treg/Th17 balance [37]. In our study, the increased serum leptin concentration could also contribute to the enhanced Th17 response, suggested by the increased serum IL-17A levels in obese mice [38]. However, this effect could not be attributed to cytokines involved in Th17 cell differentiation and maintenance, such as IL-6 and IL-1β [38], because their concentrations were not significantly increased in serum of our obese mice.

In our study, the reduction of serum pro-inflammatory cytokines in the HFD+Bif group could also be related to the parallel reduction of leptin concentrations, which could be an additional mechanism by which the bifidobacteria reduce systemic inflammation. For example, leptin increases pro-inflammatory cytokine secretion (e.g. TNF-α) by mouse monocyte/macrophages [39] as it could be the case for our study. The increased leptin levels found in diet-induced obese mice can contribute to shift the T lymphocyte subpopulations and the cytokine pool, increasing IFN-γ secretion for example in the adipose tissue [39]. Leptin has also been reported to be inversely related to Tregs numbers since it negatively controls their proliferative/survival capacity [5] and, accordingly, we detected an inverse relationship between both parameters in the peripheral circulation.

### *B*. *pseudocatenulatum* CECT 7765 contributes to reducing systemic inflammation by reducing the HFD-induced microbiota alterations and components of adaptive and innate immunity in the gut

In our study we confirm that HFD-induced gut microbiota alterations involve increases in Firmicutes and decreases in Bacteroidetes, as reported in other animal studies [19,31,40,41,42]. We also detected that the HFD increased the proportions of the phylum Proteobacteria in partial agreement with a recent study reporting increases in Enterobacteriaceae, which belong to the phylum Proteobacteria [19]. The HFD-induced reductions in the candidate division TM7 and in Verrucomicrobia reported by Shim (2013) were also supported by our data, although these phyla represent a minor proportion (<1%) of all reads.

Our study also shows that *B*. *pseudocatenulatum* CECT 7765 administration to obese mice led to partial restoration of the microbiota composition towards the one found in the control group. This restoring effect was most pronounced for the Firmicutes phylotypes; this is revealed by the fact that the abundances of the three most prominent genera and groups belonging to this phylum (unclassified Lachnospiraceae, *Allobaculum* and unclassified Erysipelotrichaceae), did not differ significantly between the the SD and the HFD+Bif groups. However, alterations in the proportions of Bacteroidetes phylotypes found in the HFD group were not restored to those found in the SD group by bifidobacteria administration. Some of these specific alterations in the microbiota had been reported in other studies. *B*. *pseudocatenulatum* CECT 7765 administration to the obese mice increased *Allobaculum* abundance to levels similar as those found in the SD and the SD+Bif groups. This is in agreement with a study applying berberine to treat obesity and diabetes in HFD-fed rats where *Allobaculum* and *Blautia* were also reversibly shifted [43]. A study on genetically obese mice (*db/db*) also reports significantly increased abundances of *Alistipes* and *Oscillibacter*, as well as exclusive presence of Delta-Proteobacteria, *Rikenella* and *Odoribacter* in obese animals, by contrast these groups were less abundant and absent, respectively, in lean animals [44]. All these taxa were also significantly increased in our HFD group and lowered by *B*. *pseudocatenulatum* CECT 7765 administration.

The resilience of the indigenous mouse microbiota to modifications by *B*. *pseudocatenulatum* CECT 7765 administration was clearly higher in control mice than in obese mice, suggesting that a disease-associated microbiota is more susceptible to probiotic intervention. However, not all potentially probiotic bacteria have demonstrated the ability to mediate effects via modulation of the gut ecosystem in mouse models of obesity, as was the case for *L*. *reuteri* ATCC [37] and *A*. *muciniphila* MucT [30], which would suggest different modes of action.

*B*. *pseudocatenulatum* CECT 7765 also seems to play a role in reducing LPS translocation in obese mice by restoration of the HFD-induced microbiota alterations, which can contribute to reducing the luminal LPS content and/or the microbiota-induced increases in intestinal permeability and inflammatory tone. The correlations between the HFD-induced increases in Proteobacteria and serum LPS levels suggest a direct contribution of the obese microbiota to increased LPS concentrations. This mechanism is also supported by the ability of *B*. *pseudocatenulatum* CECT 7765 administration to reduce the HFD-induced increases of Gram-negative bacteria, including *Escherichia/Shigella* and Desulfovibrionaceae, which can be possible sources of LPS. *B*. *pseudocatenulatum* CECT 7765 also reduced intestinal inflammatory markers (TNF-α) increased in obese mice that cause increases in paracellular permeability in different disease models [45], constituting an alternative mechanism by which it could reduce LPS translocation. Increasing intestinal bifidobacterial numbers by administering prebiotics has also been reported to reduce LPS circulating levels via improvement of the gut barrier function mediated by increased GLP-2 production and modulation of expression and localization of tight junction-related proteins [46]. However, the effects of *A*. *muciniphila* Muc^T^ in reduction of serum LPS levels and intestinal permeability have not been consistent across studies. While Everard et al. (2011) [31] reported increases in the thickness of the mucus layer and reductions in metabolic endotoxemia related to improvements in gut barrier function, Shin et al. (2014) [30] did not report improvements in intestinal permeability in diet-induced obese mice.

*B*. *pseudocatenulatum* CECT 7765 also seems to contribute to reducing inflammatory signals and systemic circulation of inflammatory mediators, generated from the activated epithelia and the gut-associated lymphoid immune system by saturated fat and the HFD-induced microbiota alterations. *B*. *pseudocatenulatum* CECT 7765 reduced HFD-induced TLR4 expression, which could amplify the innate immunity responses in the gut of obese mice [47]. In our study, TLR4 expression could be increased directly by the HFD and/or indirectly by the HFD-induced microbiota alterations. Nevertheless, a recent study reported that the HFD-induced colonic inflammation required gut colonization by the microbiota [11] and that it was partially mediated by TLR4 signalling [19]. In obesity mouse models, TLR4 is known to be involved in the inflammatory response that culminates in insulin resistance and metabolic dysfunction as demonstrated when comparing wild-type to CD14−/− mice [46], and to TLR4−/− mice [19].

*B*. *pseudocatenulatum* CECT 7765 also influenced intestinal immune-cell distribution and related cytokines, thereby being able to exert systemic effects via the cross-talk between the gut and the periphery. In particular, the administration of the bacterial strain reduced the HFD-increased intestinal B cell numbers parallel to their reductions in peripheral blood. However, intestinal Treg numbers were not influenced by the bifidobacteria, suggesting that intestinal changes in B cells are those primarily driving the effects also detected in peripheral blood, where B cell populations were also decreased parallel to increases in Treg numbers.

*B*. *pseudocatenulatum* CECT 7765 also reduced intestinal cytokine production, influencing all HFD-induced chemokines and cytokines of innate and adaptive immunity (TNF-α, MCP-1, IP-10, IL-18, IL-4, IL-6 and IL-10). A reduction in TLR expression induced by the bifidobacteria could partly explain the local reduction of cytokine production mediated by TLR-signalling. These include TNF-α, IL-1β and IL-6 whose increases in obese mice are known to be dependent on TLR4 signalling [19,48]. Increases in adaptive immunity cytokines in obese mice could be explained by the increased CD8+/CD4+ ratio and B cells with pro-inflammatory phenotype, which were at least partially reversed by *B*. *pseudocatenulatum* CECT 7765 together with related cytokines such as IFN-γ that could be produced by CD8+ and B cells and, in turn, activate secretion of other inflammatory mediators such as IP-10 and TNF-α by other immune cells (monocytes, macrophages, etc.).

Although a direct effect of the diet on gut inflammatory markers cannot be disregarded since it is known that long-chain fatty acids increase lymphocyte flux and enhance its proliferative response in intestinal lymph from mesenteric lymph duct [49], the fact that the intervention with *B*. *pseudocatenulatum* reversed the HFD-inflammatory effects suggests a primary role for the gut microbiota in the regulation of this process. Our study also supports the notion that the gut constitutes the primary origin of inflammation associated with obesity and metabolic dysfunction as expression of pro-inflammatory mediators were increased to a larger extent in the gut than in any other tissue. Furthermore, all inflammatory cytokines/chemokines significantly increased in the gut were also increased in some of the peripheral tissues studied, whereas not all these inflammatory markers were increased in every tissue of obese mice. These findings may be a logical consequence of being the place firstly exposed to this “noxious” dietary stimuli (“saturated fat”) and where there is an important load of microbes, which could contribute to inflammation under a HFD, and the largest mass of lymphoid tissue in the human body. In particular, *Bifidobacterium* spp. negatively correlated to IL-6 (R = -0.716, p = 0.020), which is a cytokine that was only significantly increased in the gut and could contribute to the increased Th17 response seen in our model parallel to B cell increases and Tregs depletion described in obese mice. The gut could also be an important source of MCP-1 that could contribute to monocyte migration and macrophage infiltration in obesity, since its concentrations were markedly increased in this tissue while not in the liver. These findings also suggest a causative role of gut inflammation in obesity and metabolic dysfunction as suggested by other authors [11,19] and could lead to reconsider the generally accepted notion that adipose tissue inflammation is the primary origin of inflammation related to metabolic dysfunction in obesity [50]. Our findings also indicate that intervention in the gut ecosystem with potentially probiotic bacteria could reduce the obesity-associated inflammation; however, effects and modes of action differ depending on the bacterial strain.

## Conclusions

The present study demonstrates that intervention in the murine gut ecosystem with *B*. *pseudocatenulatum* CECT 7765 modulates the HFD-induced immune cell infiltration and inflammation in the gut and the periphery, parallel to improvements in metabolic dysfunction associated with obesity. The study also shows for the first time that the anti-inflammatory effects exerted by this bifidobacterial strain implicate components of both innate and adaptive immunity, involving B lymphocytes. Moreover, our results support the notion that inflammatory responses originating in the gut contribute to peripheral tissue inflammation associated with HFD-induced obesity rather than being a secondary consequence of obesity and lipid accumulation in the periphery. Furthermore, intervention in the gut ecosystem with *B*. *pseudocatenulatum* CECT 7765 leading to changes in microbiota structure was sufficient to shift the inflammatory profile of the gut and beyond, proving that gut signalling plays a primary role in the regulation of obesity-associated inflammation.

## Supporting Information

S1 File Adipocyte cell size in epididymal adipose tissue (Table) and distribution of adipocytes size in epididymal adipose tissue (Figure) in different mouse groups after 14 weeks of intervention. (DOC)

S2 File Hepatic steatosis graded according to lipid accumulation in hepatocytes (Table). and examples of histologic analysis of hepatic steatosis in different mouse groups after 14 weeks of intervention (Figure). (DOC)

S3 File Bacterial abundances relative to all reads (%) obtained by pyrosequencing (Table). (XLSX)

S4 File Sequence reads assigned to *Bifidobacterium* spp. with the RDP multiclassifier detected in faeces of different mouse groups (Figure). (PNG)

## References

[1] Ng M, Fleming T, Robinson M, Thomson B, Graetz N, Margono C, et al (2014) Global, regional, and national prevalence of overweight and obesity in children and adults during 1980–2013: a systematic analysis for the Global Burden of Disease Study 2013. Lancet 384(9945):766–81. 10.1016/S0140-6736(14)60460-8 24880830 PMC4624264

[2] Wolowczuk I, Verwaerde C, Viltart O, Delanoye A, Delacre M, Pot B, et al (2008) Feeding our immune system: impact on metabolism. Clin Dev Immunol 2008:639803 10.1155/2008/639803 18350123 PMC2266987

[3] Kanneganti TD, Dixit VD (2012) Immunological complications of obesity. Nat Immunol 13 (8):707–12. 10.1038/ni.2343 22814340

[4] Olefsky JM, Glass CK (2010) Macrophages, inflammation, and insulin resistance. Annu Rev Physiol 72:219–46. 10.1146/annurev-physiol-021909-135846 20148674

[5] Feuerer M, Hill JA, Mathis D, Benoist C (2009) Foxp3+ regulatory T cells: differentiation, specification, subphenotypes. A review. Nat Immunol 10(7):689–95. 10.1038/ni.1760 19536194

[6] Nishimura S, Manabe I, Nagasaki M, Eto K, Yamashita H, Ohsugi M, et al (2009) CD8+ effector T cells contribute to macrophage recruitment and adipose tissue inflammation in obesity. Nat Med 15(8):914–20. 10.1038/nm.1964 19633658

[7] DeFuria J, Belkina AC, Jagannathan-Bogdan M, Snyder-Cappione J, Carr JD, Nersesova YR, et al (2013) B cells promote inflammation in obesity and type 2 diabetes through regulation of T-cell function and an inflammatory cytokine profile. Proc Natl Acad Sci U S A. 110(13):5133–8. 10.1073/pnas.1215840110 23479618 PMC3612635

[8] Li H, Lelliott C, Hakansson P, Ploj K, Tuneld A, Verolin-Johansson M, et al (2008) Intestinal, adipose, and liver inflammation in diet-induced obese mice. Metabolism 57: 1704–1710. 10.1016/j.metabol.2008.07.029 19013294

[9] Gerner RR, Wieser V, Moschen AR, Tilg H (2013) Metabolic inflammation: role of cytokines in the crosstalk between adipose tissue and liver. Can J Physiol Pharmacol 91(11):867–72. 10.1139/cjpp-2013-0050 24117253

[10] Paul G, Schäffler A, Neumeier M, Fürst A, Bataillle F, Buechler C, et al (2006) Profiling adipocytokine secretion from creeping fat in Crohn's disease. Inflamm Bowel Dis 12:471–7 16775490 10.1097/00054725-200606000-00005

[11] Ding S, Chi MM, Scull BP, Rigby R, Schwerbrock NM, Magness S, et al (2010) High-fat diet: bacteria interactions promote intestinal inflammation which precedes and correlates with obesity and insulin resistance in mouse. PLoS One 16;5(8):e12191 10.1371/journal.pone.0012191 PMC2922379 20808947

[12] Goel A, Gupta M, Aggarwal R (2014) Gut microbiota and liver disease. J Gastroenterol Hepatol 29(6):1139–48. 10.1111/jgh.12556 24547986

[13] Li XM, Jeffers LJ, Reddy KR, de Medina M, Silva M, Villanueva S, et al (1991) Immunophenotyping of lymphocytes in livertissue of patients with chronicliver diseases by flow cytometry. Hepatology 14(1):121–7. 1712336 10.1002/hep.1840140120

[14] Borst SE, Conover CF (2005) High-fat diet induces increased tissue expression of TNF-alpha. Life Sci 77(17):2156–65. 15935403 10.1016/j.lfs.2005.03.021

[15] Sanz Y, Rastmanesh R, Agostoni C (2013) Understanding the role of gut microbes and probiotics in obesity: how far are we? Pharmacol Res 69(1):144–55. 10.1016/j.phrs.2012.10.021 23147032

[16] Cani PD, Delzenne NM (2009) Interplay between obesity and associated metabolic disorders: new insights into the gut microbiota. Curr Opin Pharmacol 9(6):737–743. 10.1016/j.coph.2009.06.016 19628432

[17] Cano PG, Santacruz A, Trejo FM, Sanz Y (2013) *Bifidobacterium* CECT 7765 improves metabolic and immunological alterations associated with obesity in high-fat diet fed mice. Obesity (Silver Spring) 21(11):2310–21. 23418126 10.1002/oby.20330

[18] Arndt T, Jörns A, Weiss H, Tiedge M, Hedrich HJ, Lenzen S, et al (2013) A Variable CD3(+) T-Cell Frequency in Peripheral Blood Lymphocytes Associated with Type 1 Diabetes Mellitus Development in the LEW.1AR1-iddm Rat. PLoS One 8(5):e64305 10.1371/journal.pone.0064305 23717591 PMC3661438

[19] Kim KA, Gu W, Lee IA, Joh EH, Kim DH (2012) High fat diet-induced gut microbiota exacerbates inflammation and obesity in mice via the TLR4 signaling pathway. PLoS One 7(10):e47713 10.1371/journal.pone.0047713 23091640 PMC3473013

[20] Sim K, Cox MJ, Wopereis H, Martin R, Knol J, Li MS, et al (2012) Improved detection of bifidobacteria with optimised 16S rRNA-gene based pyrosequencing. PLoS One 7 (3):e32543 10.1371/journal.pone.0032543 22470420 PMC3314643

[21] Schloss PD, Westcott SL, Ryabin T, Hall JR, Hartmann M, Hollister EB, et al (2009) Introducing mothur: Open-source, platform-independent, community-supported software for describing and comparing microbial communities. Appl Environ Microbiol 75(23):7537–41. 10.1128/AEM.01541-09 19801464 PMC2786419

[22] Wang Q, Garrity GM, Tiedje JM, Cole JR (2007) Naïve Bayesian Classifier for Rapid Assignment of rRNA Sequences into the New Bacterial Taxonomy. Appl Environ Microbiol 73(16):5261–7 17586664 10.1128/AEM.00062-07 PMC1950982

[23] Caporaso JG, Kuczynski J, Stombaugh J, Bittinger K, Bushman FD, Costello EK, et al (2010) QIIME allows analysis of high-throughput community sequencing data. Nat Methods 7(5):335–6. 10.1038/nmeth.f.303 20383131 PMC3156573

[24] Matsuki T, Watanabe K, Fujimoto J, Miyamoto Y, Takada T, Matsumoto K, et al (2002) Development of 16S rRNA-gene-targeted group-specific primers for the detection and identification of predominant bacteria in human feces. Appl Environ Microbiol 68: 5445–5451. 12406736 10.1128/AEM.68.11.5445-5451.2002 PMC129894

[25] Matsuki T, Watanabe K, Fujimoto J, Takada T, Tanaka R (2004) Use of 16S rRNA gene-targeted group-specific primers for real-time PCR analysis of predominant bacteria in human feces. Appl Environ Microbiol 70(12):7220–8. 15574920 10.1128/AEM.70.12.7220-7228.2004 PMC535136

[26] Santacruz A, Collado MC, Garcia-Valdes L, Segura MT, Martin-Lagos JA, Anjos T, et al (2010) Gut microbiota composition is associated with body weight, weight gain and biochemical parameters in pregnant women. Br J Nutr 104: 83–92. 10.1017/S0007114510000176 20205964

[27] Duffaut C, Zakaroff-Girard A, Bourlier V, Decaunes P, Maumus M, Chiotasso P, et al (2009) Interplay between human adipocytes and T lymphocytes in obesity: CCL20 as an adipochemokine and T lymphocytes as lipogenic modulators. Arterioscler Thromb Vasc Biol. 29(10):1608–14. 10.1161/ATVBAHA.109.192583 19644053

[28] Winer DA, Winer S, Shen L, Wadia PP, Yantha J, Paltser G, et al (2011) B cells promote insulin resistance through modulation of T cells and production of pathogenic IgG antibodies. Nat Med 7(5):610–7. 10.1038/nm.2353 PMC3270885 21499269

[29] Winer DA, Winer S, Shen L, Chng MH, Engleman EG (2012) B lymphocytes as emerging mediators of insulin resistance.Int J Obes Suppl 2 (Suppl 1):S4–S7. 10.1038/ijosup.2012.2 PMC4109086 25089193

[30] Shin NR, Lee JC, Lee HY, Kim MS, Whon TW, Lee MS, et al (2014) An increase in the *Akkermansia* spp. population induced by metformin treatment improves glucose homeostasis in diet-induced obese mice. Gut 63(5):727–35. 10.1136/gutjnl-2012-303839 23804561

[31] Everard A, Lazarevic V, Derrien M, Girard M, Muccioli GG, Neyrinck AM, et al (2011) Responses of gut microbiota and glucose and lipid metabolism to prebiotics in genetic obese and diet-induced leptin-resistant mice. Diabetes 60(11):2775–86. 10.2337/db11-0227 21933985 PMC3198091

[32] Ma X, Hua J, Li Z (2008) Probiotics improve high fat diet-induced hepatic steatosis and insulin resistance by increasing hepatic NKT cells. J Hepatol 49(5):821–30. 10.1016/j.jhep.2008.05.025 18674841 PMC2588670

[33] Esposito E, Iacono A, Bianco G, Autore G, Cuzzocrea S, Vajro P, et al (2009) Probiotics reduce the inflammatory response induced by a high-fat diet in the liver of young rats. J Nutr 139(5):905–11. 10.3945/jn.108.101808 19321579

[34] Obstfeld AE, Sugaru E, Thearle M, Francisco AM, Gayet C, Ginsberg HN, et al (2010) C-C chemokine receptor 2 (CCR2) regulates the hepatic recruitment of myeloid cells that promote obesity-induced hepatic steatosis.Diabetes 59(4):916–25. 10.2337/db09-1403 20103702 PMC2844839

[35] Oh DY, Morinaga H, Talukdar S, Bae EJ, Olefsky JM (2012) Increased macrophage migration into adipose tissue in obese mice. Diabetes 61(2):346–54. 10.2337/db11-0860 22190646 PMC3266418

[36] Schlegel PM, Steiert I, Kötter I, Müller CA (2013) B Cells Contribute to Heterogeneity of IL-17 Producing Cells in Rheumatoid Arthritis and Healthy Controls. PLoS One 8(12):e82580 10.1371/journal.pone.0082580 24340045 PMC3855537

[37] Poutahidis T, Kleinewietfeld M, Smillie C, Levkovich T, Perrotta A, Bhela S, et al (2013) Microbial reprogramming inhibits Western diet-associated obesity. PLoS One 8(7):e68596 10.1371/journal.pone.0068596 23874682 PMC3707834

[38] Ericksen RE, Rose S, Westphalen CB, Shibata W, Muthupalani S, Tailor Y, et al (2014) Obesity accelerates *Helicobacter felis*-induced gastric carcinogenesis by enhancing immature myeloid cell trafficking and TH17 response. Gut 63(3):385–94. 10.1136/gutjnl-2013-305092 23729675 PMC3972255

[39] Matarese G, Moschos S, Mantzoros CS (2005) Leptin in immunology. A review. J Immunol 174(6):3137–42. 15749839 10.4049/jimmunol.174.6.3137

[40] Turnbaugh PJ, Backhed F, Fulton L, Gordon JI (2008) Diet-induced obesity in linked to marked but reversible alterations in the mouse distal gut microbiome. Cell Host Microbe 3(4):213–23. 10.1016/j.chom.2008.02.015 18407065 PMC3687783

[41] Turnbaugh PJ, Ridaura VK, Faith JJ, Rey FE, Knight R, Gordon JI (2009) The effect of diet on the human gut microbiome: a metagenomic analysis in humanized gnotobiotic mice. Sci Transl Med 1(6):6ra14 10.1126/scitranslmed.3000322 20368178 PMC2894525

[42] Shim JO (2013) Gut Microbiota in Inflammatory Bowel Disease. Pediatr Gastroenterol Hepatol Nutr 16(1):17–21. 10.5223/pghn.2013.16.1.17 24010101 PMC3746045

[43] Zhang X, Zhao Y, Zhang M, Pang X, Xu J, Kang C, et al (2012) Structural changes of gut microbiota during berberine-mediated prevention of obesity and insulin resistance in high-fat diet-fed rats. PLoS One 7(8):e42529 10.1371/journal.pone.0042529 22880019 PMC3411811

[44] Geurts L, Lazarevic V, Derrien M, Everard A, Van Roye M, Knauf C, et al (2011) Altered gut microbiota and endocannabinoid system tone in obese and diabetic leptin-resistant mice: impact on apelin regulation in adipose tissue. Front. Microbiol 13;2:149. 10.3389/fmicb.2011.00149 PMC3139240 21808634

[45] Corridoni D, Pastorelli L, Mattioli B, Locovei S, Ishikawa D, Arseneau KO, et al (2012) Probiotic bacteria regulate intestinal epithelial permeability in experimental ileitis by a TNF-dependent mechanism. PLoS One 7(7):e42067 10.1371/journal.pone.0042067 22848704 PMC3405026

[46] Cani PD, Neyrinck AM, Fava F, Knauf C, Burcelin RG, Tuohy KM, et al (2007) Selective increases of bifidobacteria in gut microflora improve high-fat-diet-induced diabetes in mice through a mechanism associated with endotoxaemia. Diabetologia 50: 2374–2383. 17823788 10.1007/s00125-007-0791-0

[47] Sanz Y, De Palma G (2009) Gut microbiota and probiotics in modulation of epithelium and gut-associated lymphoid tissue function. Int Rev Immunol 28(6):397–413. 10.3109/08830180903215613 19954356

[48] Jialal I, Kaur H, Devaraj S (2014) Toll-like receptor status in obesity and metabolic syndrome: a translational perspective. J Clin Endocrinol Metab 99(1):39–48. 10.1210/jc.2013-3092 24187406

[49] Miura S, Imaeda H, Shiozaki H, Ohkubo N, Tashiro H, Serizawa H, et al (1993) Increased proliferative response of lymphocytes from intestinal lymph during long chain fatty acid absorption. Immunology 78(1):142–6. 8436400 PMC1421772

[50] Maury E, Brichard SM (2010) Adipokine dysregulation, adipose tissue inflammation and metabolic syndrome. Mol Cell Endocrinol 314: 1–16. 10.1016/j.mce.2009.07.031 19682539

