# Supplementary material for: Bifidobacterium pseudocatenulatum CECT 7765 Reduces Obesity-Associated Inflammation by Restoring the Lymphocyte-Macrophage Balance and Gut Microbiota Structure in High-Fat Diet-Fed Mice
Source: PLoS One. 2015 Jul 10;10(7):e0126976. doi: 10.1371/journal.pone.0126976 (PMC4498624; doi:10.1371/journal.pone.0126976)
Supplement: S1 File — (DOC) [file pone.0126976.s001.doc]

**S1A Table. Adipocyte cell size in epididymal adipose tissue in different mouse groups after 14 weeks of intervention**

| **% Adipocyte cell sizes (µm^2^)** | **Experimental Groups** | | | | | | |  |
| --- | --- | --- | --- | --- | --- | --- | --- | --- |
|  | **SD** | **HFD** | **SD+Bif** | **HFD+Bif** | ***P*- value**  (HFD vs. SD) | ***P*- value**  (SD+ Bif vs. SD) | ***P*- value**  (HFD+ Bif vs. HFD) | ***P*- value**  (HFD+ Bif vs. SD) |

Mean  *se*  Mean  *se* Mean *se*  Mean *se*

| <2000 | 76.27 | 20.26 | 0.00 | 0.00 | 65.22 | 20.83 | 10.17 | 5.80 | <0.001* | 0.490 | 0.006* | 0.007* |
| --- | --- | --- | --- | --- | --- | --- | --- | --- | --- | --- | --- | --- |
| 2000-4000 | 24.01 | 9.71 | 13.10 | 2.20 | 34.76 | 20.89 | 53.43 | 1.57 | 0.008* | 0.858 | <0.001* | 0.145 |
| 4000-6000 | 0.00 | 0.00 | 40.63 | 16.10 | 0.00 | 0.00 | 32.47 | 5.51 | 0.008* | >0.999 | 0.365 | 0.011* |
| >6000 | 0.00 | 0.00 | 52.05 | 8.26 | 0.00 | 0.00 | 4.19 | 0.80 | <0.001* | >0.999 | 0.005* | 0.067 |

SD group: control mice receiving a SD plus placebo; HFD group: obese mice receiving a HFD plus placebo; SD+Bif group: control mice receiving SD and a daily dose of 1 x10^9^ CFU *B. pseudocatenulatum* CECT 7765; HFD+Bif group: obese mice receiving HFD and a daily dose of 1 x10^9^ CFU *B. pseudocatenulatum* CECT 7765 by gavage during 14 weeks (n=10). Adipocyte cell sizes are expressed as area ranges subdivided as follows: <2000, 2000-4000, 4000-6000 and 6000- 7000 µm2. *Significant differences were established by ANOVA and *post hoc* Bonferroni’s test at *p≤*0.050.


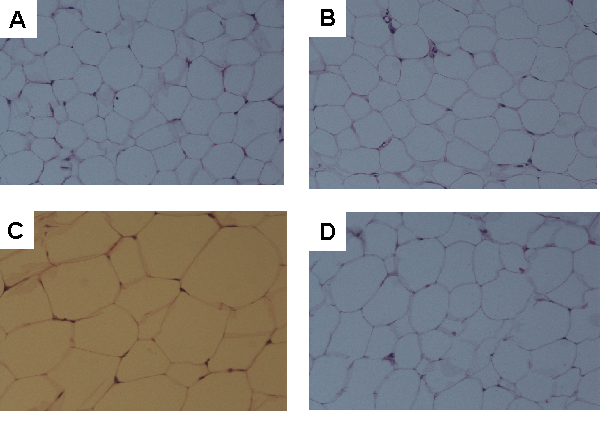


**S1B Figure**. **Distribution of adipocytes size in epididymal adipose tissue** **in different mouse groups after 14 weeks of intervention.** Photomicrographs 20X of representative HE-stained slides are shown**. Image A:** Control mice receiving SD plus placebo (SD group) (n=10); **Image B**: Control mice receiving SD plus a daily dose of 1 x10^9^ CFU *B. pseudocatenulatum* CECT 7765 (SD+Bif group) by gavage during 14 weeks (n=10); **Image C**: Obese mice receiving HFD plus placebo (HFD group) (n=10); **Image D**: Obese mice receiving HFD plus a daily dose of 1 x10^9^ CFU *B. pseudocatenulatum* CECT 7765 (HFD+Bif group) by gavage during 14 weeks (n=10).
