# Supplementary material for: Bifidobacterium pseudocatenulatum CECT 7765 Reduces Obesity-Associated Inflammation by Restoring the Lymphocyte-Macrophage Balance and Gut Microbiota Structure in High-Fat Diet-Fed Mice
Source: PLoS One. 2015 Jul 10;10(7):e0126976. doi: 10.1371/journal.pone.0126976 (PMC4498624; doi:10.1371/journal.pone.0126976)
Supplement: S2 File — (DOC) [file pone.0126976.s002.doc]

**S2 Table. Hepatic steatosis graded according to lipid accumulation in hepatocytes in different mouse groups after 14 weeks of intervention**

| **Steatosis (%)** | **Experimental Groups** | | | | | | |  |
| --- | --- | --- | --- | --- | --- | --- | --- | --- |
|  | **SD** | **HFD** | **SD+Bif** | **HFD+Bif** | ***P*- value**  (HFD vs. SD) | ***P*- value**  (SD+ Bif vs. SD) | ***P*- value**  (HFD+ Bif vs. HFD) | ***P*- value**  (HFD+ Bif vs. SD) |

Mean  *se*  Mean  *se* Mean *se*  Mean *se*

| Grade 0 | 36.05 | 19.25 | 0.00 | 0.00 | 49.91 | 26.50 | 55.29 | 5.03 | 0.004* | 0.482 | <0.001* | 0.212 |
| --- | --- | --- | --- | --- | --- | --- | --- | --- | --- | --- | --- | --- |
| Grade 1 | 37.75 | 6.69 | 13.69 | 11.00 | 25.32 | 16.67 | 44.71 | 5.03 | 0.055 | 0.285 | 0.016* | 0.272 |
| Grade 2 | 26.18 | 21.00 | 43.88 | 13.94 | 25.05 | 4.48 | 0.00 | 0.00 | 0.195 | 0.947 | 0.002* | 0.095 |
| Grade 3 | 0.00 | 0.00 | 43.21 | 26.40 | 0.00 | 0.00 | 0.00 | 0.00 | 0.043* | >0.999 | 0.043* | >0.999 |

SD group: control mice receiving a SD plus placebo; HFD group: obese mice receiving a HFD plus placebo; SD+Bif group: control mice receiving SD and a daily dose of 1 x10^9^ CFU *B. pseudocatenulatum* CECT 7765; HFD+Bif group: obese mice receiving HFD and a daily dose of 1 x10^9^ CFU *B. pseudocatenulatum* CECT 7765 by gavage during 14 weeks (n=10). The fat vacuoles were measured in 100 hepatocytes of two liver tissue sections per mouse and scored for the severity of steatosis according to the following criteria: for grade-0 steatosis, no fatty hepatocytes; grade-1 steatosis, fat occupying less than 30% of the hepatocyte; grade-2 steatosis, fat occupying less than 30 to 60 % of the hepatocyte; grade-3 steatosis, fat occupying more than 60 % of the hepatocyte. *Significant differences were established by ANOVA and post hoc Bonferroni’s test at p≤0.050.


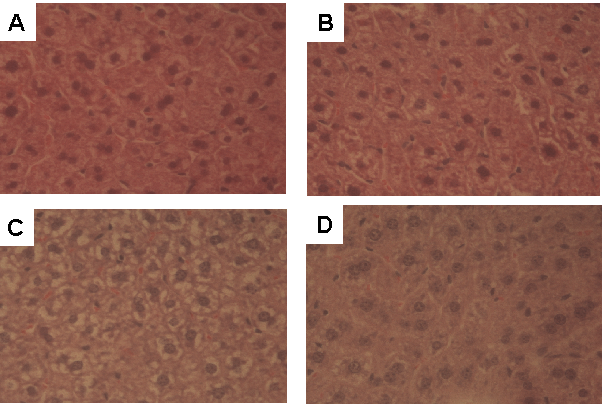


**S2 Fig. Determination of hepatic steatosis** **in different mouse groups after 14 weeks of intervention.** Photomicrographs 20X of representative HE-stained slides are shown**. Image A:** Control mice receiving SD plus placebo (SD group) (n=10); **Image B**: Control mice receiving SD plus a daily dose of 1 x10^9^ CFU *B. pseudocatenulatum* CECT 7765 (SD+Bif group) by gavage during 14 weeks (n=10); **Image C**: Obese mice receiving HFD plus placebo (HFD group) (n=10); **Image D**: Obese mice receiving HFD plus a daily dose of 1 x10^9^ CFU *B. pseudocatenulatum* CECT 7765 (HFD+Bif group) by gavage during 14 weeks (n=10).
