# Supplementary figures and images for: Bifidobacterium pseudocatenulatum CECT 7765 Reduces Obesity-Associated Inflammation by Restoring the Lymphocyte-Macrophage Balance and Gut Microbiota Structure in High-Fat Diet-Fed Mice
Source: PLoS One. 2015 Jul 10;10(7):e0126976. doi: 10.1371/journal.pone.0126976 (PMC4498624; doi:10.1371/journal.pone.0126976)

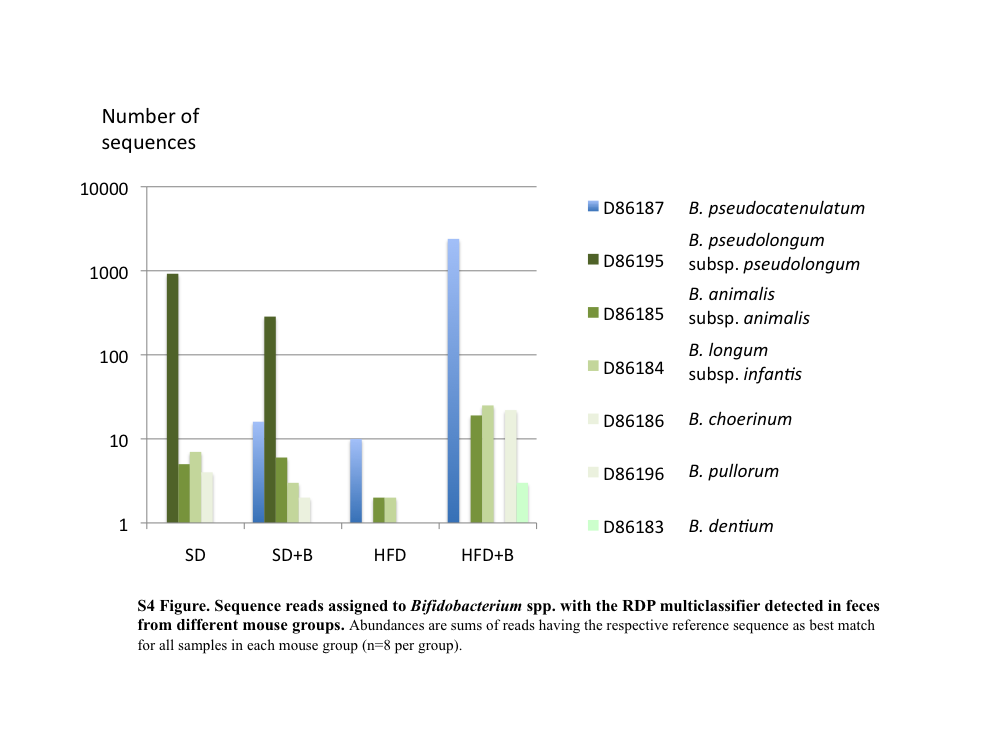

Supplement: S4 File — (PNG) [file pone.0126976.s004.png]
